# Increased mutation and gene conversion within human segmental duplications

**DOI:** 10.1038/s41586-023-05895-y

**Published:** 2023-05-10

**Authors:** Mitchell R. Vollger, Philip C. Dishuck, William T. Harvey, William S. DeWitt, Xavi Guitart, Michael E. Goldberg, Allison N. Rozanski, Julian Lucas, Mobin Asri, Human Pangenome Reference Consortium, Haley J. Abel, Lucinda L. Antonacci-Fulton, Gunjan Baid, Carl A. Baker, Anastasiya Belyaeva, Konstantinos Billis, Guillaume Bourque, Silvia Buonaiuto, Andrew Carroll, Mark J. P. Chaisson, Pi-Chuan Chang, Xian H. Chang, Haoyu Cheng, Justin Chu, Sarah Cody, Vincenza Colonna, Daniel E. Cook, Robert M. Cook-Deegan, Omar E. Cornejo, Mark Diekhans, Daniel Doerr, Peter Ebert, Jana Ebler, Jordan M. Eizenga, Susan Fairley, Olivier Fedrigo, Adam L. Felsenfeld, Xiaowen Feng, Christian Fischer, Paul Flicek, Giulio Formenti, Adam Frankish, Robert S. Fulton, Yan Gao, Shilpa Garg, Erik Garrison, Nanibaa’ A. Garrison, Carlos Garcia Giron, Richard E. Green, Cristian Groza, Andrea Guarracino, Leanne Haggerty, Ira M. Hall, Marina Haukness, David Haussler, Simon Heumos, Glenn Hickey, Thibaut Hourlier, Kerstin Howe, Miten Jain, Erich D. Jarvis, Hanlee P. Ji, Eimear E. Kenny, Barbara A. Koenig, Alexey Kolesnikov, Jan O. Korbel, Jennifer Kordosky, Sergey Koren, HoJoon Lee, Heng Li, Wen-Wei Liao, Shuangjia Lu, Tsung-Yu Lu, Julian K. Lucas, Hugo Magalhães, Santiago Marco-Sola, Pierre Marijon, Charles Markello, Tobias Marschall, Fergal J. Martin, Ann McCartney, Jennifer McDaniel, Karen H. Miga, Matthew W. Mitchell, Jean Monlong, Jacquelyn Mountcastle, Moses Njagi Mwaniki, Maria Nattestad, Adam M. Novak, Sergey Nurk, Hugh E. Olsen, Nathan D. Olson, Benedict Paten, Trevor Pesout, Adam M. Phillippy, Alice B. Popejoy, Pjotr Prins, Daniela Puiu, Mikko Rautiainen, Allison A. Regier, Arang Rhie, Samuel Sacco, Ashley D. Sanders, Valerie A. Schneider, Baergen I. Schultz, Kishwar Shafin, Jonas A. Sibbesen, Jouni Sirén, Michael W. Smith, Heidi J. Sofia, Ahmad N. Abou Tayoun, Françoise Thibaud-Nissen, Chad Tomlinson, Francesca Floriana Tricomi, Flavia Villani, Mitchell R. Vollger, Justin Wagner, Brian Walenz, Ting Wang, Jonathan M. D. Wood, Aleksey V. Zimin, Justin M. Zook, Katherine M. Munson, Alexandra P. Lewis, Kendra Hoekzema, Glennis A. Logsdon, David Porubsky, Benedict Paten, Kelley Harris, PingHsun Hsieh, Evan E. Eichler

**Affiliations:** 1 Department of Genome Sciences, University of Washington School of Medicine, Seattle, WA USA; 2 Division of Medical Genetics, University of Washington School of Medicine, Seattle, WA USA; 3 Computational Biology Program, Fred Hutchinson Cancer Research Center, Seattle, WA USA; 4 Department of Electrical Engineering and Computer Sciences, University of California, Berkeley, Berkeley, CA USA; 5 UC Santa Cruz Genomics Institute, University of California, Santa Cruz, Santa Cruz, CA USA; 6 Howard Hughes Medical Institute, Chevy Chase, MD USA; 7 Division of Oncology, Department of Internal Medicine, Washington University School of Medicine, St Louis, MO USA; 8 McDonnell Genome Institute, Washington University School of Medicine, St Louis, MO USA; 9 Google LLC, Mountain View, CA USA; 10 European Molecular Biology Laboratory, European Bioinformatics Institute, Hinxton, UK; 11 Department of Human Genetics, McGill University, Montreal, Quebec Canada; 12 Canadian Center for Computational Genomics, McGill University, Montreal, Quebec Canada; 13 Institute for the Advanced Study of Human Biology (WPI-ASHBi), Kyoto University, Kyoto, Japan; 14 Institute of Genetics and Biophysics, National Research Council, Naples, Italy; 15 Department of Quantitative and Computational Biology, University of Southern California, Los Angeles, CA USA; 16 Department of Data Sciences, Dana-Farber Cancer Institute, Boston, MA USA; 17 Department of Biomedical Informatics, Harvard Medical School, Boston, MA USA; 18 Department of Genetics, Genomics and Informatics, University of Tennessee Health Science Center, Memphis, TN USA; 19 Barrett and O’Connor Washington Center, Arizona State University, Washington DC, USA; 20 Department of Ecology and Evolutionary Biology, University of California, Santa Cruz, Santa Cruz, CA USA; 21 Institute for Medical Biometry and Bioinformatics, Medical Faculty, Heinrich Heine University Düsseldorf, Düsseldorf, Germany; 22 Center for Digital Medicine, Heinrich Heine University Düsseldorf, Düsseldorf, Germany; 23 Core Unit Bioinformatics, Medical Faculty, Heinrich Heine University Düsseldorf, Düsseldorf, Germany; 24 Vertebrate Genome Laboratory, The Rockefeller University, New York, NY USA; 25 National Institutes of Health (NIH)–National Human Genome Research Institute, Bethesda, MD USA; 26 Department of Genetics, Washington University School of Medicine, St Louis, MO USA; 27 Center for Computational and Genomic Medicine, The Children’s Hospital of Philadelphia, Philadelphia, PA USA; 28 Novo Nordisk Foundation Center for Biosustainability, Technical University of Denmark, Copenhagen, Denmark; 29 Institute for Society and Genetics, College of Letters and Science, University of California, Los Angeles, Los Angeles, CA USA; 30 Institute for Precision Health, David Geffen School of Medicine, University of California, Los Angeles, Los Angeles, CA USA; 31 Division of General Internal Medicine and Health Services Research, David Geffen School of Medicine, University of California, Los Angeles, Los Angeles, CA USA; 32 Department of Biomolecular Engineering, University of California, Santa Cruz, Santa Cruz, CA USA; 33 Dovetail Genomics, Scotts Valley, CA USA; 34 Quantitative Life Sciences, McGill University, Montreal, Quebec Canada; 35 Genomics Research Centre, Human Technopole, Milan, Italy; 36 Department of Genetics, Yale University School of Medicine, New Haven, CT USA; 37 Center for Genomic Health, Yale University School of Medicine, New Haven, CT USA; 38 Quantitative Biology Center (QBiC), University of Tübingen, Tübingen, Germany; 39 Biomedical Data Science, Department of Computer Science, University of Tübingen, Tübingen, Germany; 40 Tree of Life, Wellcome Sanger Institute, Hinxton, UK; 41 Northeastern University, Boston, MA USA; 42 Laboratory of Neurogenetics of Language, The Rockefeller University, New York, NY USA; 43 Division of Oncology, Department of Medicine, Stanford University School of Medicine, Stanford, CA USA; 44 Institute for Genomic Health, Icahn School of Medicine at Mount Sinai, New York, NY USA; 45 Program in Bioethics and Institute for Human Genetics, University of California, San Francisco, San Francisco, CA USA; 46 Genome Biology Unit, European Molecular Biology Laboratory, Heidelberg, Germany; 47 Genome Informatics Section, Computational and Statistical Genomics Branch, National Human Genome Research Institute, National Institutes of Health, Bethesda, MD USA; 48 Division of Biology and Biomedical Sciences, Washington University School of Medicine, St Louis, MO USA; 49 Computer Sciences Department, Barcelona Supercomputing Center, Barcelona, Spain; 50 Departament d’Arquitectura de Computadors i Sistemes Operatius, Universitat Autònoma de Barcelona, Barcelona, Spain; 51 Material Measurement Laboratory, National Institute of Standards and Technology, Gaithersburg, MD USA; 52 Coriell Institute for Medical Research, Camden, NJ USA; 53 Department of Computer Science, University of Pisa, Pisa, Italy; 54 Department of Public Health Sciences, University of California, Davis, Davis, CA USA; 55 Department of Biomedical Engineering, Johns Hopkins University, Baltimore, MD USA; 56 Department of Ecology and Evolutionary Biology, University of California, Santa Cruz, Santa Cruz, CA USA; 57 Berlin Institute for Medical Systems Biology, Max Delbrück Center for Molecular Medicine in the Helmholtz Association, Berlin, Germany; 58 National Center for Biotechnology Information, National Library of Medicine, National Institutes of Health, Bethesda, MD USA; 59 Center for Health Data Science, University of Copenhagen, Copenhagen, Denmark; 60 Al Jalila Genomics Center of Excellence, Al Jalila Children’s Specialty Hospital, Dubai, United Arab Emirates; 61 Center for Genomic Discovery, Mohammed Bin Rashid University of Medicine and Health Sciences, Dubai, United Arab Emirates; 62 Center for Computational Biology, Johns Hopkins University, Baltimore, MD USA

**Keywords:** Genetic variation, Genome evolution, Mutation, Structural variation, Evolutionary genetics

## Abstract

Single-nucleotide variants (SNVs) in segmental duplications (SDs) have not been systematically assessed because of the limitations of mapping short-read sequencing data^1,2^. Here we constructed 1:1 unambiguous alignments spanning high-identity SDs across 102 human haplotypes and compared the pattern of SNVs between unique and duplicated regions^3,4^. We find that human SNVs are elevated 60% in SDs compared to unique regions and estimate that at least 23% of this increase is due to interlocus gene conversion (IGC) with up to 4.3 megabase pairs of SD sequence converted on average per human haplotype. We develop a genome-wide map of IGC donors and acceptors, including 498 acceptor and 454 donor hotspots affecting the exons of about 800 protein-coding genes. These include 171 genes that have ‘relocated’ on average 1.61 megabase pairs in a subset of human haplotypes. Using a coalescent framework, we show that SD regions are slightly evolutionarily older when compared to unique sequences, probably owing to IGC. SNVs in SDs, however, show a distinct mutational spectrum: a 27.1% increase in transversions that convert cytosine to guanine or the reverse across all triplet contexts and a 7.6% reduction in the frequency of CpG-associated mutations when compared to unique DNA. We reason that these distinct mutational properties help to maintain an overall higher GC content of SD DNA compared to that of unique DNA, probably driven by GC-biased conversion between paralogous sequences^5,6^.

## Abstract (web summary)

A study comparing the pattern of single-nucleotide variation between unique and duplicated regions of the human genome shows that mutation rate and interlocus gene conversion are elevated in duplicated regions.

## Main

The landscape of human SNVs has been well characterized for more than a decade in large part owing to wide-reaching efforts such as the International HapMap Project and the 1000 Genomes Project^7,8^. Although these consortia helped to establish the genome-wide pattern of SNVs (as low as 0.1% allele frequency) and linkage disequilibrium on the basis of sequencing and genotyping thousands of human genomes, not all parts of the human genome could be equally ascertained. Approximately 10–15% of the human genome^8^ has remained inaccessible to these types of analysis either because of gaps in the human genome sequence or, more frequently, the low mapping quality associated with aligning short-read whole-genome sequencing data. This is because short-read sequence data are of insufficient length (<200 base pairs (bp)) to unambiguously assign reads and, therefore, variants to specific loci^9^. Although certain classes of large, highly identical repeats (for example, α-satellites in centromeres) were readily recognized, others, especially SDs^1^ and their 859 associated genes^10^, in euchromatin were much more problematic to recognize.

Operationally, SDs are defined as interchromosomal or intrachromosomal homologous regions in any genome that are >1 kbp in length and >90% identical in sequence^1,11^. As such regions arise by duplication as opposed to retrotransposition, they were initially difficult to identify and early versions of the human genome sequence had either missed or misassembled these regions owing to their high sequence identity^12,13^. Large-insert BAC clones ultimately led to many of these regions being resolved. Subsequent analyses showed that SDs contribute disproportionately to copy number polymorphisms and disease structural variation^9,14^, are hotspots for gene conversion^15^, are substantially enriched in GC-rich DNA and Alu repeats^16,17^, and are transcriptionally diverse leading to the emergence, in some cases, of human-specific genes thought to be important for human adaptation^18–21^. Despite their importance, the pattern of SNVs among humans has remained poorly characterized. Early on, paralogous sequence variants were misclassified as SNVs^2^ and, as a result, later high-identity SDs became blacklisted from SNV analyses because short-read sequence data could not be uniquely placed^22,23^. This exclusion has translated into a fundamental lack of understanding in mutational processes precisely in regions predicted to be more mutable owing to the action of IGC^24–28^. Previously, we noted an increase in SNV density in duplicated regions when compared to unique regions of the genome on the basis of our comparison of GRCh38 and the complete telomere-to-telomere (T2T) human reference genome^10^. Leveraging high-quality phased genome assemblies from 47 humans generated as part of the Human Pangenome Reference Consortium (HPRC)^3^, we sought to investigate this difference more systematically and compare the SNV landscape of duplicated and unique DNA in the human genome revealing distinct mutational properties.

## Strategy and quality control

**Extended Data Fig. 1:**
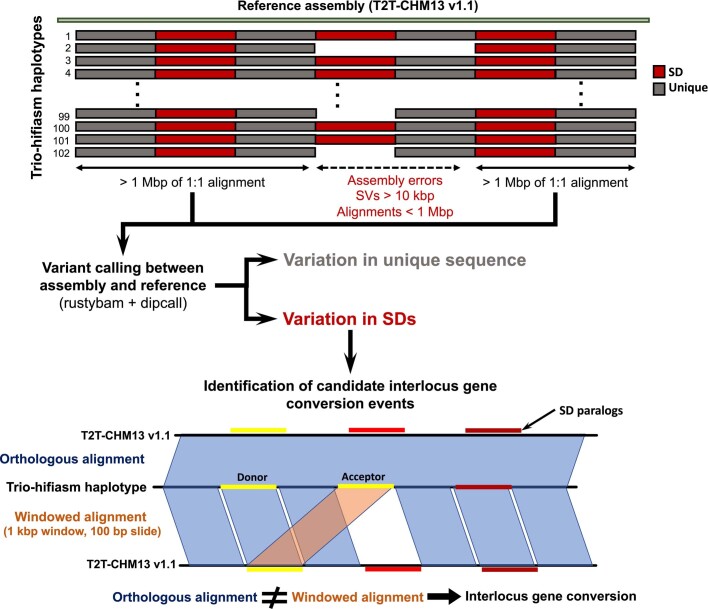
Analysis schema for variant and IGC calling. Whole-genome alignments were calculated for the HPRC assemblies against T2T-CHM13 v1.1 with a copy of GRCh38 chrY using minimap2 v2.24. The alignments were further processed to remove alignments that were redundant in query sequence or that had structural variants over 10 kbp in length. After these steps, the remaining alignments over 1 Mbp were defined to be syntenic and used in downstream analyses. We then counted all pairwise single-nucleotide differences between the haplotypes and the reference and stratified these results into unique regions versus SD regions based on the SD annotations from T2T-CHM13 v1.1. All variants intersecting tandem repeats were filtered to avoid spurious SNV calls. To detect candidate regions of IGC, the query sequence with syntenic alignments was fragmented into 1 kbp windows with a 100 bp slide and realigned back to T2T-CHM13 v1.1 independent of the flanking sequence using minimap2 v2.24 to identify each window’s single best alignment position. These alignments were compared to their original syntenic alignment positions, and if they were not overlapping, we considered them to be candidate IGC windows. Candidate IGC windows were then merged into larger intervals and realigned when windows were overlapping in both the donor and the acceptor sequence. We then used the CIGAR string to identify the number of matching and mismatching bases at the “donor” site and compared that to the number of matching and mismatching bases at the acceptor site determined by the syntenic alignment to calculate the number of supporting SNVs.

Unlike previous SNV discovery efforts, which catalogued SNVs on the basis of the alignment of sequence reads, our strategy was assembly driven (Extended Data Fig. 1). We focused on the comparison of 102 haplotype-resolved genomes (Supplementary Table 1) generated as part of the HPRC (*n* = 94) or other efforts (*n* = 8)^3,4,12,29^ in which phased genome assemblies had been assembled using high-fidelity (HiFi) long-read sequencing^30^. The extraordinary assembly contiguity of these haplotypes (contig N50, defined as the sequence length of the shortest contig at 50% of the total assembly length, > 40 Mbp) provided an unprecedented opportunity to align large swathes (>1 Mbp) of the genome, including high-identity SD repeats anchored by megabases of synteny.

As SD regions are often enriched in assembly errors even among long-read assemblies^3,4,31^, we carried out a series of analyses to assess the integrity and quality of these regions in each assembled haplotype. First, we searched for regions of collapse^11^ by identifying unusual increases or decreases in sequence read depth^3^. We determine that, on average, only 1.64 Mbp (1.37%) of the analysed SD sequence was suspect owing to unusually high or low sequence read depth on the basis of mapping of underlying read data— as such patterns are often indicative of a misassembly^3^ (Methods). Next, for all SD regions used in our analysis we compared the predicted copy number by Illumina sequence read depth with the sum based on the total copy number from the two assembled haplotypes. These orthogonal copy number estimates were highly correlated (Pearson’s *R*  = 0.99, *P* < 2.2 × 10^−16^; Supplementary Fig. 1) implying that most SD sequences in the assemblies have the correct copy number. To confirm these results in even the most difficult to assemble duplications, we selected 19 of the largest and most identical SDs across 47 haplotypes for a total of 893 tests. These estimates were also highly correlated (Pearson’s *R* = 0.99, *P* < 2.2 × 10^−16^; Supplementary Figs. 2 and 3), and of the 893 tests conducted, 756 were identical. For the 137 tests for which estimates differed, most (*n* = 125) differed by only one copy. Finally, most of these discrepancies came from just three large (>140 kbp) and highly identical (>99.3%) SDs (Supplementary Fig. 3).

**Extended Data Fig. 2:**
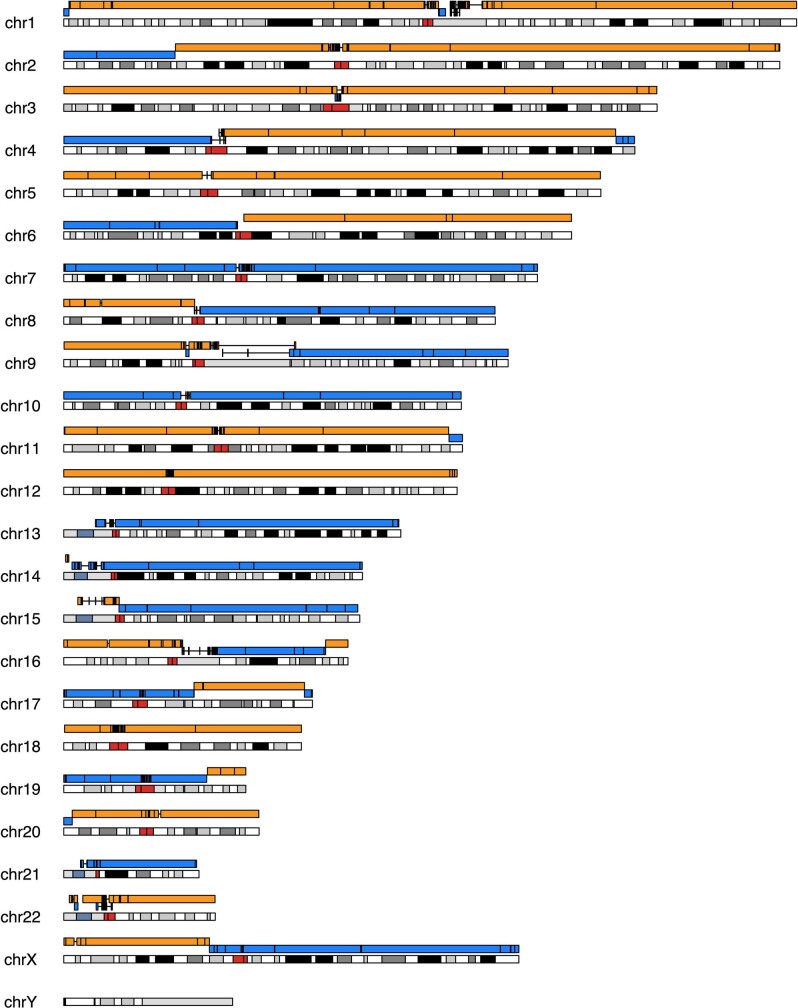
Ideogram of an assembly of CHM1 aligned to T2T-CHM13. The ideogram depicts the contiguity (alternating blue and orange contigs) of a CHM1 assembly generated by Verkko as compared to T2T-CHM13. The overall contig N50 is 105.2 Mbp providing near chromosome arm contiguity with the exception of breaks at the centromere (red) and other large satellite arrays. Because the sequence is derived from a monoploid complete hydatidiform mole, there is no opportunity for assembly errors due to inadvertent haplotype switching.

To validate the base-level accuracy, we next compared the quality value for both SD and unique sequences using Illumina sequencing data for 45 of the HPRC samples (Methods). Both unique (average quality value = 59 s.d. 1.9) and SD (average quality value = 53 s.d. 1.9) regions are remarkably high quality, which in the case of SDs translates into less than 1 SNV error every 200 kbp (Supplementary Fig. 4). We further show that these high-quality assembles result in accurate variant calls (Supplementary Notes and Supplementary Figs. 5–9). We also assessed the contiguity of the underlying assemblies using a recently developed tool, GAVISUNK, which compares unique *k*-mer distributions between HiFi-based assemblies and orthogonal Oxford Nanopore Technologies sequencing data from the same samples. We found that, on average, only 0.11% of assayable SD sequence was in error compared to 0.14% of unique regions assayed (Supplementary Table 2), implying high and comparable assembly contiguity. As a final control for potential haplotype-phasing errors introduced by trio HiFi assembly of diploid samples, we generated deep Oxford Nanopore Technologies and HiFi data from a second complete hydatidiform mole (CHM1) for which a single paternal haplotype was present and applied a different assembly algorithm^32^ (Verkko 1.0; Extended Data Fig. 2). We show across our many analyses that the results from the CHM1 Verkko assembly are consistent with individual haplotypes obtained from diploid HPRC samples produced by trio hifiasm^3,32^ (Supplementary Fig. 10). We therefore conclude that phasing errors have, at most, a negligible effect on our results and that most (>98%) SDs analysed were accurately assembled from multiple human genomes allowing the pattern of SNV diversity in SDs to be systematically interrogated.

## Increased SNV density in SD regions

**Fig. 1:**
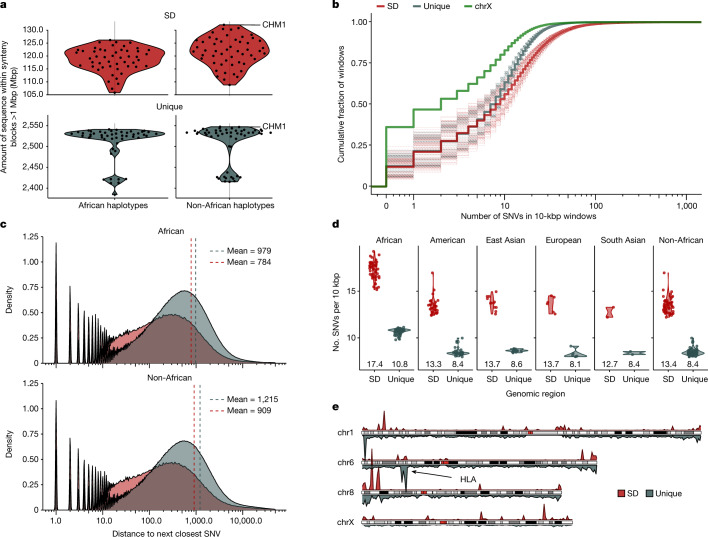
Increased single-nucleotide variation in SDs. **a**, The portion of the human genome analysed for SD (red) and unique (blue) regions among African and non-African genomes. Shown are the number of megabase pairs aligned in 1:1 syntenic blocks to T2T-CHM13 v1.1 for each assembled haplotype. Data are shown as both a single point per haplotype originating from a single individual and a smoothed violin plot to represent the population distribution. **b**, Empirical cumulative distribution showing the number of SNVs in 10-kbp windows in the syntenic regions stratified by unique (grey), SD (red) and the X chromosome (chrX; green). Dashed lines represent individual haplotypes and thick lines represent the average trend of all the data. **c**, Distribution of the average distance to the next closest SNV in SD (red) and unique (grey) space separating African (top) and non-African (bottom) samples. Dashed vertical lines are drawn at the mean of each distribution. **d**, Average number of SNVs per 10-kbp window in SD (red) versus unique (grey) space by superpopulation and with mean value shown underneath each violin. The non-African column represents an aggregation of the data from all non-African populations in this study. **e**, Density of SNVs in 10 bp of each other for SD (top, red) and unique (bottom, grey) regions for chromosomes 1, 6, 8 and X comparing the relative density of known (for example, HLA) and new hotspots of single-nucleotide variation.

**Extended Data Fig. 3:**
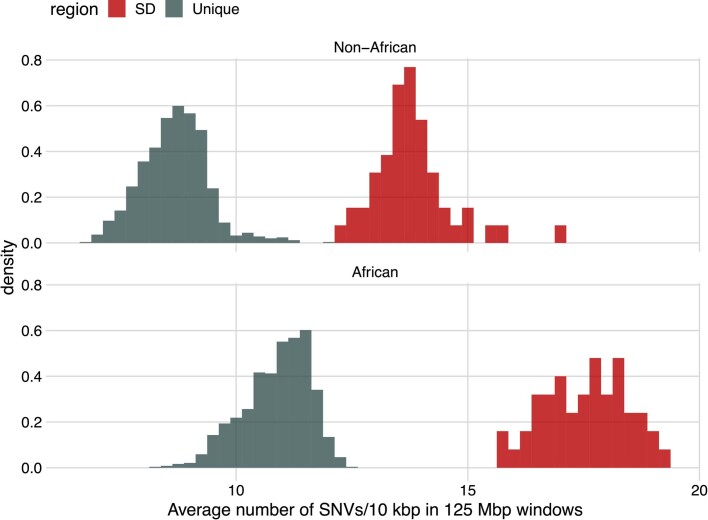
Increased variation in SD sequences and African haplotypes. Histograms of the average number of SNVs per 10 kbp over all 125 Mbp bins of unique (blue) and SD (red) sequence for all haplotypes. African haplotypes (bottom) are compared separately to non-African (top) haplotypes. All SD bins (125 Mbp each) have more SNVs than any unique bin irrespective of human superpopulation.

**Extended Data Fig. 4:**
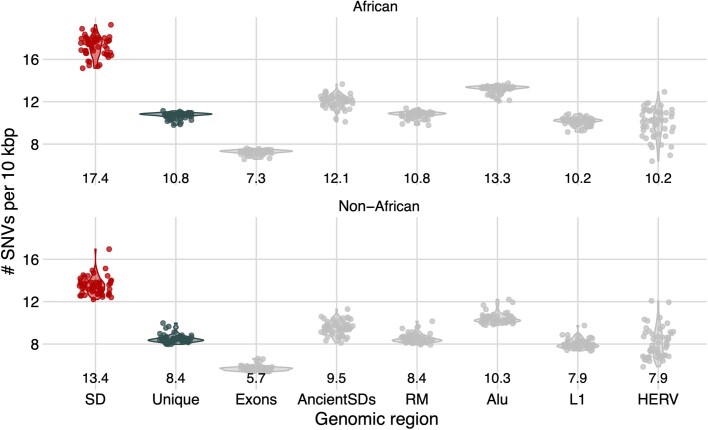
Average number of SNVs across different repeat classes. Shown are the average number of SNVs per 10 kbp within SDs (red), unique (blue), and additional sequence classes (gray) across the HPRC haplotypes. These classes include exonic regions, ancient SDs (SD with <90% sequence identity) and all elements identified by RepeatMasker (RM) with Alu, L1 LINE, and HERV elements broken out separately. Below each sequence class we show the average number of SNVs per 10 kbp for the median haplotype. Standard deviations and measurements for additional repeat classes are provided in Table S3.

To assess SNVs, we limited our analysis to portions of the genome where a 1:1 orthologous relationship could be unambiguously assigned (as opposed to regions with extensive copy number variation). Using the T2T-CHM13 reference genome, we aligned the HPRC haplotypes requiring alignments to be a minimum of 1 Mbp in length and carry no structural variation events greater than 10 kbp (Methods and Extended Data Fig. 1). Although the proportion of haplotypes compared for any locus varied (Fig. 1a), the procedure allowed us to establish, on average, 120.2 Mbp 1:1 fully aligned sequence per genome for SD regions out of a total of 217 Mbp from the finished human genome (T2T-CHM13 v1.1). We repeated the analysis for ‘unique’ (or single-copy) regions of the genome and recovered by comparison 2,508 Mbp as 1:1 alignments (Fig. 1a). All downstream analyses were then carried out using this orthologous alignment set. We first compared the SNV diversity between unique and duplicated regions excluding suboptimal alignments mapping to tandem repeats or homopolymer stretches. Overall, we observe a significant 60% increase in SNVs in SD regions (Methods; Pearson’s chi-squared test with Yates’s continuity correction *P* < 2.2 × 10^−16^; Fig. 1b). Specifically, we observe an average of 15.3 SNVs per 10 kbp versus 9.57 SNVs per 10 kbp for unique sequences (Fig. 1d). An empirical cumulative distribution comparing the number of SNVs in 10-kbp windows between SD and unique sequence confirms that this is a general property and not driven simply by outliers. The empirical cumulative distribution shows that more than half of the SD sequences have more SNVs than their unique counterparts (Fig. 1b). Moreover, for all haplotypes we divided the unique portions of the genome into 125-Mbp bins and found that all SD bins of equivalent size have more SNVs than any of the bins of unique sequence (empirical *P* value < 0.0005; Extended Data Fig. 3). This elevation in SNVs is only modestly affected by the sequence identity of the underlying SDs (Pearson’s correlation of only 0.008; Supplementary Fig. 11). The increase in SNVs (60%) in SDs is greater than that in all other assayable classes of repeats: Alu (23%), L1 (−9.4%), human endogenous retroviruses (−9.4%) and ancient SDs for which the divergence is greater than 10% (12%) (Extended Data Fig. 4 and Supplementary Table 3). We find, however, that SNV density correlates with increasing GC content (Supplementary Fig. 12) consistent with Alu repeats representing the only other class of common repeat to show an elevation.

Previous publications have shown that African haplotypes are genetically more diverse, having on average about 20% more variant sites compared to non-African haplotypes^8^. To confirm this observation in our data, we examined the number of SNVs per 10 kbp of unique sequence in African versus non-African haplotypes (Fig. 1c,d) and observed a 27% (10.8 versus 8.5) excess in African haplotypes. As a result, among African haplotypes, we see that the average distance between SNVs (979 bp) is 19.4% closer than in non-African haplotypes (1,215 bp), as expected^8,12^. African genomes also show increased variation in SDs, but it is less pronounced with an average distance of 784 bases between consecutive SNVs as compared to 909 bases in non-African haplotypes (13.8%). Although elevated in African haplotypes, SNV density is higher in SD sequence across populations and these properties are not driven by a few sites but, once again, are a genome-wide feature. We put forward three possible hypotheses to account for this increase although note these are not mutually exclusive: SDs have unique mutational mechanisms that increase SNVs; SDs have a deeper average coalescence than unique parts of the genome; and differences in sequence composition (for example, GC richness) make SDs more prone to particular classes of mutation.

## Putative IGC

**Fig. 2:**
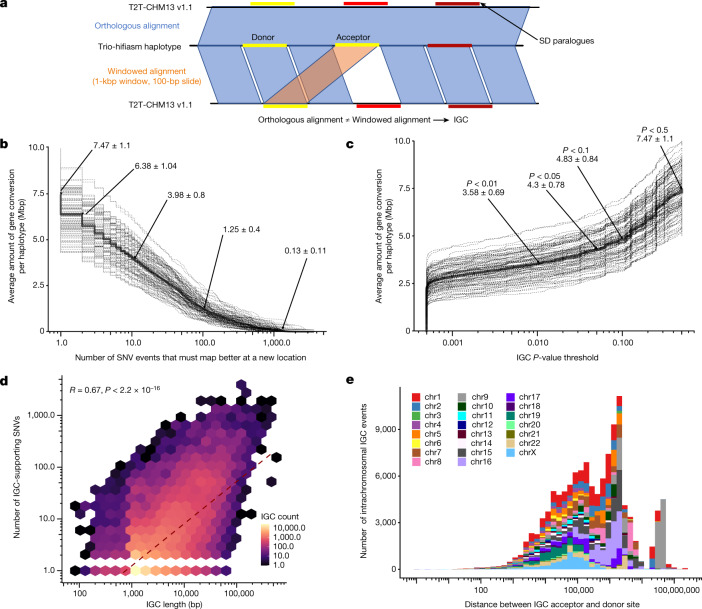
Candidate IGC events. **a**, Method to detect IGC. The assembled human haplotype query sequence from 1:1 syntenic alignments was fragmented into 1-kbp windows in 100-bp increments and realigned back to T2T-CHM13 v1.1 independent of the flanking sequence information using minimap2 v2.24 to identify each window’s single best alignment position. These alignments were compared to their original syntenic alignment positions, and if they were not overlapping, we considered them to be candidate IGC windows. Candidate IGC windows were then merged into larger intervals and realigned when windows were overlapping in both the donor and the acceptor sequence. We then used the CIGAR string to identify the number of matching and mismatching bases at the ‘donor’ site and compared that to the number of matching and mismatching bases at the acceptor site determined by the syntenic alignment to calculate the number of supporting SNVs. **b**, The amount of SDs (in megabase pairs) predicted to be affected by IGC per haplotype, as a function of the minimum number of SNVs that support the IGC call. Dashed lines represent individual haplotypes and the solid line represents the average. **c**, Empirical cumulative distribution of the megabase pairs of candidate IGC observed in HPRC haplotypes, as a function of the minimum underlying *P*-value threshold used to define the IGC callset (see Methods for IGC *P*-value calculation). Dashed lines represent individual haplotypes and the solid line represents the average. **d**, Correlation between IGC length and the number of supporting SNVs. **e**, Distribution of the distance between predicted IGC acceptor and donor sites for intrachromosomal events by chromosome.

One possible explanation for increased diversity in SDs is IGC in which sequence that is orthologous by position no longer shares an evolutionary history because a paralogue from a different location has ‘donated’ its sequence through ectopic template-driven conversion^33^, also known as nonallelic gene conversion^27^. To identify regions of IGC, we developed a method that compares two independent alignment strategies to pinpoint regions where the orthologous alignment of an SD sequence is inferior to an independent alignment of the sequence without flanking information (Fig. 2a and Methods). We note several limitations of our approach (Supplementary Notes); however, we show that our high-confidence IGC calls (20+ supporting SNVs) have strong overlap with other methods for identifying IGC (Supplementary Notes and Supplementary Fig. 13). Using this approach, we created a genome-wide map of putative large IGC events for all of the HPRC haplotypes for which 1:1 orthologous relationships could be established (Fig. 2).

**Extended Data Fig. 5:**
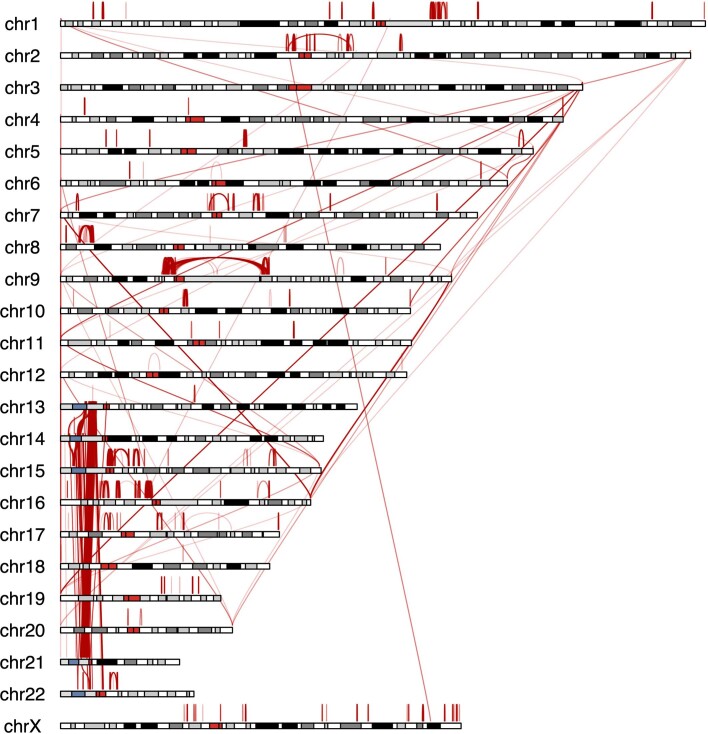
Largest IGC events in the human genome. The ideogram depicts as red arcs the positions of the largest IGC events between and within human chromosomes (top 10% of the length distribution).

Across all 102 haplotypes, we observe 121,631 putative IGC events for an average of 1,193 events per human haplotype (Fig. 2b,c and Supplementary Table 4). Of these events, 17,949 are rare and restricted to a single haplotype (singletons) whereas the remaining events are observed in several human haplotypes grouping into 14,663 distinct events (50% reciprocal overlap at both the donor and acceptor site). In total, we estimate that there is evidence for 32,612 different putative IGC events (Supplementary Table 5) among the SD regions that are assessed at present. Considering the redundant IGC callset (*n* = 121,631), the average IGC length observed in our data is 6.26 kbp with the largest event observed being 504 kbp (Extended Data Fig. 5). On average, each IGC event has 13.3 SNVs that support the conversion event and 2.03 supporting SNVs per kilobase pair, and as expected, there is strong correlation (Pearson’s *R* = 0.63, *P* < 2.2 × 10^−16^; Fig. 2d) between the length of the events and supporting SNVs. Furthermore, we validated these supporting SNVs against Illumina sequencing data and find that on average only 1% (12/1,192) of IGC events contain even one erroneous SNV (Supplementary Fig. 4). The putative IGC events detected with our method are largely restricted to higher identity duplications with only 325 events detected in 66.1 Mbp of SDs with >10% sequence divergence (Supplementary Figs. 14 and 15). We further stratify these results by callset, minimum number of supporting SNVs and haplotype (Supplementary Table 6). Finally, we use the number of supporting informative SNVs to estimate the statistical confidence of every putative IGC call (Fig. 2c, Supplementary Table 7 and Methods). Using these *P* values, we identify a subset of the high-confidence (*P* value < 0.05) IGC calls with 31,910 IGC events and 10,102 nonredundant events.

**Fig. 3:**
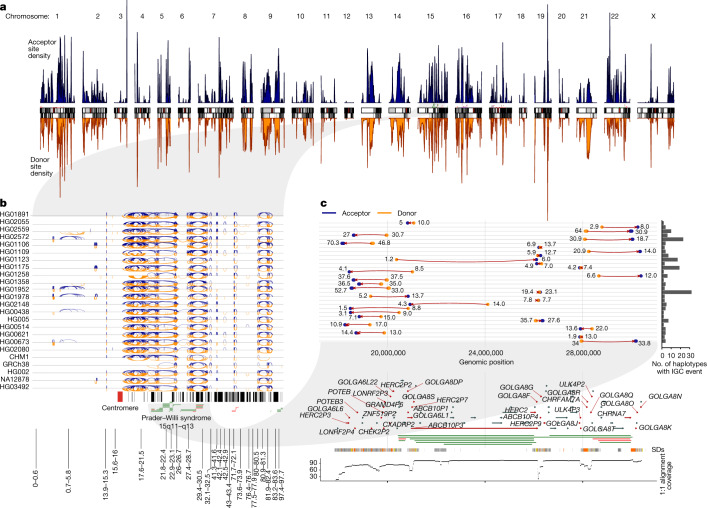
IGC hotspots. **a**, Density of IGC acceptor (top, blue) and donor (bottom, orange) sites across the ‘SD genome’. The SD genome consists of all main SD regions (>50 kbp) minus the intervening unique sequences. **b**, All intrachromosomal IGC events on 24 human haplotypes analysed for chromosome 15. Arcs drawn in blue (top) have the acceptor site on the left-hand side and the donor site on the right. Arcs drawn in orange (bottom) are arranged oppositely. Protein-coding genes are drawn as vertical black lines above the ideogram, and large duplication (blue) and deletion (red) events associated with human diseases are drawn as horizontal lines just above the ideogram. **c**, Zoom of the 30 highest confidence (lowest *P* value) IGC events on chromosome 15 between 17 and 31 Mbp. The number to the left of each event shows its length (kbp) and that to the right shows its number of SNVs. Genes with IGC events are highlighted in red and associate with the breakpoint regions of Prader–Willi syndrome. An expanded graphic with all haplotypes is included in Extended Data Fig. 7.

On average, we identify 7.5 Mbp of sequence per haplotype affected by putative IGC and 4.3 Mbp in our high-confidence callset (Fig. 2b). Overall, 33.8% (60.77/180.0 Mbp) of the analysed SD sequence is affected by putative IGC in at least one human haplotype. Furthermore, among all SDs covered by at least 20 assembled haplotypes, we identify 498 acceptor and 454 donor IGC hotspots with at least 20 distinct IGC events (Fig. 3 and Supplementary Table 8). IGC hotspots are more likely to associate with higher copy number SDs compared to a random sample of SD windows of equal size (median of 9 overlaps compared to 3, one-sided Wilcoxon rank sum test *P* < 2.2 × 10^−16^) and regions with more IGC events are moderately correlated with the copy number of the SD (Pearson’s *R* = 0.23, *P* < 2.2 × 10^−16^; Supplementary Fig. 16). IGC hotspots also preferentially overlap higher identity duplications (median 99.4%) compared to randomly sampled windows (median 98.0%, one-sided Wilcoxon rank sum test *P* < 2.2 × 10^−16^).

**Fig. 4:**
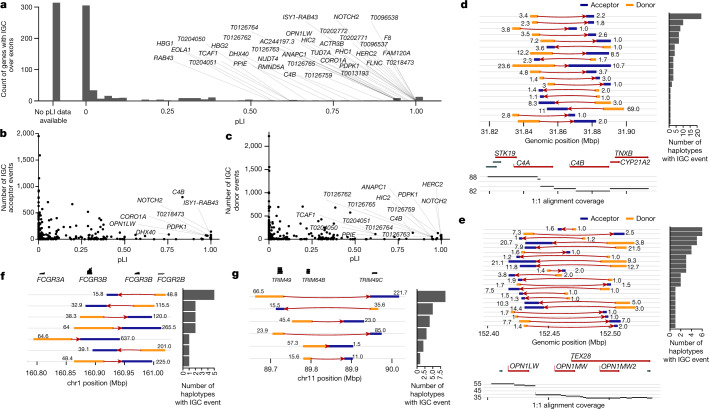
Protein-coding genes affected by IGC. **a**, Number of putative IGC events intersecting exons of protein-coding genes as a function of a gene’s pLI. Of the 799 genes, 314 (39.3%) did not have a pLI score and are shown in the column labelled No pLI data available. **b**,**c**, Number of times a gene exon acts as an acceptor (**b**) or a donor (**c**) of an IGC event. **d**,**e**, IGC events at the complement factor locus, *C4A* and *C4B* (**d**), and the opsin middle- and long-wavelength-sensitive genes associated with colour blindness (*OPN1MW* and *OPN1LW* locus; **e**). Predicted donor (orange) and acceptor (blue) segments by length (number to left of event) and average number of supporting SNVs (number to right of event) are shown. The number of human haplotypes supporting each configuration is depicted by the histograms to the right. **f**,**g**, IGC events that reposition entire gene models for the *FCGR* (**f**) and *TRIM* (**g**) loci.

These events intersect 1,179 protein-coding genes, and of these genes, 799 have at least one coding exon affected by IGC (Supplementary Tables 9 and 10). As a measure of functional constraint, we used the probability of being loss-of-function intolerant (pLI) for each of the 799 genes^34^ (Fig. 4a). Among these, 314 (39.3%) have never been assessed for mutation intolerance (that is, no pLI) owing to the limitations of mapping short-read data from population samples^34^. Of the remaining genes, we identify 38 with a pLI greater than 0.5, including genes associated with disease (*F8*, *HBG1* and *C4B*) and human evolution (*NOTCH2* and *TCAF*). Of the genes with high pLI scores, 12 are the acceptor site for at least 50 IGC events, including *CB4*, *NOTCH2* and *OPNL1W—*a locus for red–green colour blindness (Fig. 4b–e). We identify a subset of 418 nonredundant IGC events that are predicted to copy the entirety of a gene body to a ‘new location’ in the genome (Fig. 4f,g). As a result, 171 different protein-coding genes with at least 2 exons and 200 coding base pairs are converted in their entirety by putative IGC events in a subset of human haplotypes (Supplementary Table 11), and we refer to this phenomenon as gene repositioning. These gene-repositioning events are large (average 26 kbp; median 16.7 kbp) and supported by a high number of SNVs (average 64.7; median 15.3 SNVs), suggesting that they are unlikely to be mapping artefacts. Markedly, these putative IGC events copy the reference gene model on average a distance of 1.66 Mbp (median 216 kbp) from its original location. These include several disease-associated genes (for example, *TAOK2*, *C4A*, *C4B*, *PDPK1* and *IL27*) as well as genes that have eluded complete characterization owing to their duplicative nature^35–37^.

## Evolutionary age of SDs

**Extended Data Fig. 6:**
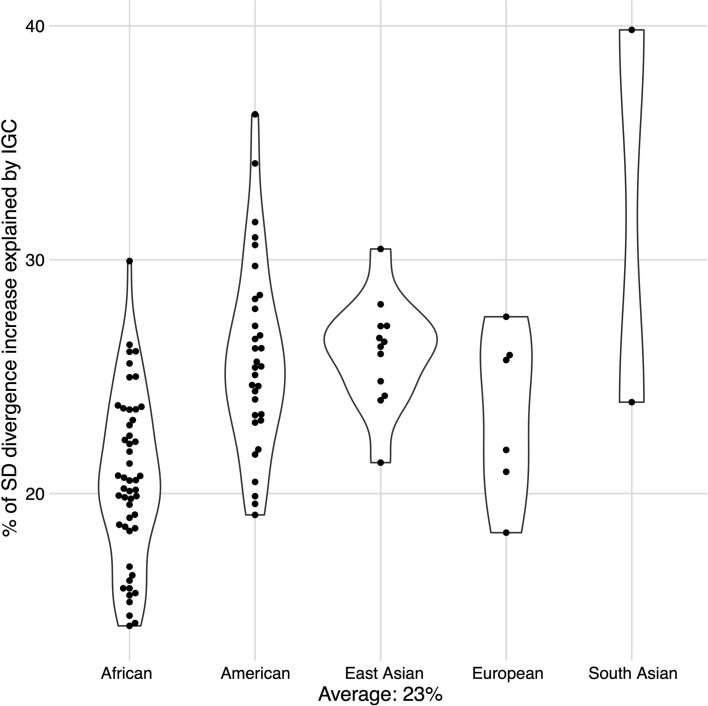
Percent of increased single-nucleotide variation explained by IGC. Shown is the fraction of the increased SNV diversity in SDs that can be attributed to IGC for each of the HPRC haplotypes stratified by global superpopulation. In text is the average across all haplotypes (23%).

Our analysis suggests that putative IGC contributes modestly to the significant increase of human SNV diversity in SDs. For example, if we apply the least conservative definition of IGC (1 supporting SNV) and exclude all putative IGC events from the human haplotypes, we estimate that it accounts for only 23% of the increase (Extended Data Fig. 6). If we restrict to higher confidence IGC events (*P* < 0.05), only 19.6% of the increase could be accounted for. An alternative explanation may be that the SDs are evolutionarily older, perhaps owing to reduced selective constraint on duplicated copies^38,39^. To test whether SD sequences seem to have a deeper average coalescence than unique regions, we constructed a high-quality, locally phased assembly (hifiasm v0.15.2) of a chimpanzee (*Pan troglodytes*) genome to calibrate age since the time of divergence and to distinguish ancestral versus derived alleles in human SD regions (Methods). Constraining our analysis to syntenic regions between human and chimpanzee genomes (Methods), we characterized 4,316 SD regions (10 kbp in size) where we had variant calls from at least 50 human and one chimpanzee haplotype. We selected at random 9,247 analogous windows from unique regions for comparison. We constructed a multiple sequence alignment for each window and estimated the time to the most recent common ancestor (TMRCA) for each 10-kbp window independently. We infer that SDs are significantly older than the corresponding unique regions of similar size (Supplementary Figs. 17 and 18; one-sided Wilcoxon rank sum test *P* value = 4.3 × 10^−14^), assuming that mutation rates have remained constant over time within these regions since the human–chimpanzee divergence. The TMRCAs inferred from SD regions are, on average, 22% more ancient when compared to unique regions (650 versus 530 thousand years ago (ka)), but only a 5% difference is noted when comparing the median (520 versus 490 ka). However, this effect all but disappears (only a 0.2% increase) after excluding windows classified as IGC (Supplementary Fig. 19; one-sided Wilcoxon rank sum test *P* = 0.05; mean TMRCA_unique_ = 528 ka, mean TMRCA_SD_ = 581 ka, median TMRCA_unique_ = 495 ka, median TMRCA_SD_ = 496 ka).

## SNV mutational spectra in SDs

**Fig. 5:**
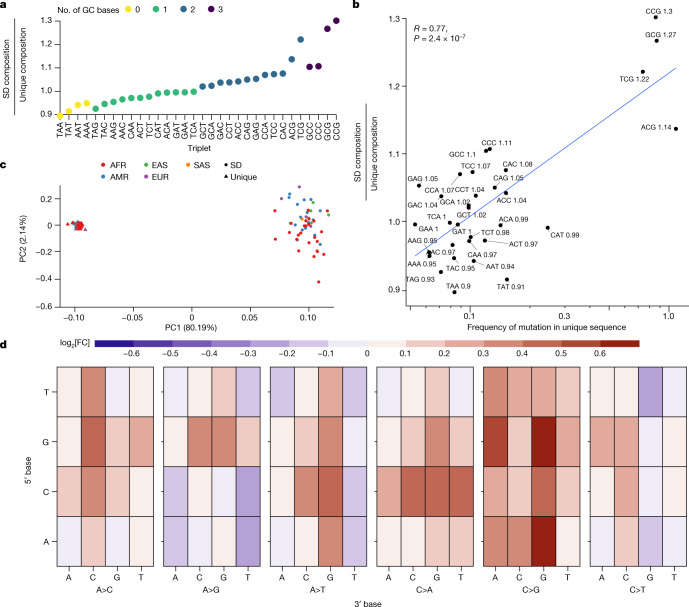
Sequence composition and mutational spectra of SD SNVs. **a**, Compositional increase in GC-containing triplets in SD versus unique regions of the genome (coloured by GC content). **b**, Correlation between the enrichment of certain triplets in SDs compared to the mutability of that triplet in unique regions of the genome. Mutability is defined as the sum of all SNVs that change a triplet divided by the total count of that triplet in the genome. The enrichment ratio of SD over unique regions is indicated in text next to each triplet sequence. The text (upper left) indicates the value of the Pearson’s correlation coefficient and the *P* value from a two-sided *t*-test without adjustment for multiple comparisons. **c**, PCA of the mutational spectra of triplets in SD (circles) versus unique (triangles) regions polarized against a chimpanzee genome assembly and coloured by the continental superpopulation of the sample. AFR, African; AMR, American; EAS, East Asian; EUR, European; SAS, South Asian. **d**, The log[fold change] in triplet mutation frequency between SD and unique sequences. The *y* axis represents the 5′ base of the triplet context; the first level of the *x* axis shows which central base has changed and the second level of the *x* axis shows the 3′ base: heatmap depicts the log[fold change]. As an example, the top left corner shows the log[fold change] in frequency of TAA>TCA mutations in SD versus unique sequences.

As a third possibility, we considered potential differences in the sequence context of unique and duplicated DNA. It has been recognized for almost two decades that human SDs are particularly biased towards Alu repeats and GC-rich DNA of the human genome^16,40^. Notably, among the SNVs in SDs, we observed a significant excess of transversions (transition/transversion ratio (Ti/Tv) = 1.78) when compared to unique sequence (Ti/Tv = 2.06; *P* < 2.2 × 10^−16^, Pearson’s chi-squared test with Yates’s continuity correction). Increased mutability of GC-rich DNA is expected and may explain, in part, the increased variation in SDs and transversion bias^6,27,41^. Using a more complete genome, we compared the GC composition of unique and duplicated DNA specifically for the regions considered in this analysis. We find that, on average, 42.4% of the analysed SD regions are guanine or cytosine (43.0% across all SDs) when compared to 40.8% of the unique DNA (*P* value < 2.2 × 10^−16^, one-sided *t*-test). Notably, this enrichment drops slightly (41.8%) if we exclude IGC regions. Consequently, we observe an increase of all GC-containing triplets in SD sequences compared to unique regions of the genome (Fig. 5a). Furthermore, the enrichment levels of particular triplet contexts in SD sequence correlate with the mutability of the same triplet sequence in unique regions of the genome (Pearson’s *R* = 0.77, *P* = 2.4 × 10^−7^; Fig. 5b). This effect is primarily driven by CpG-containing triplets, which are enriched between 14 and 30% in SD sequences. Note, we observe a weaker and insignificant correlation for the non-CpG-containing triplets (Pearson’s *R* = 0.22, *P* = 0.27). Extrapolating from the mutational frequencies seen in unique sequences, we estimate that there is 3.21% more variation with SDs due to their sequence composition alone.

To further investigate the changes in GC content and their effect on variation in SDs, we compared the triplet mutational spectra of SNVs from unique and duplicated regions of the genome to determine whether the predominant modes of SNV mutation differed (Methods). We considered all possible triplet changes, first quantifying the number of ancestral GC bases and triplets in SDs (Fig. 5a). A principal component analysis (PCA) of these normalized mutational spectra shows clear discrimination (Fig. 5c) between unique and SD regions (PC1) beyond that of African and non-African diversity, with the first principal component capturing 80.2% of the variation separating the mutational spectrum of SDs and unique DNA. We observe several differences when comparing the triplet-normalized mutation frequency of particular mutational events in SD and unique sequences (Fig. 5d). Most notable is a 7.6% reduction in CpG transition mutations—the most predominant mode of mutation in unique regions of the genome due to spontaneous deamination of methylated CpGs^6^ (Supplementary Tables 12 and 13).

The most notable changes in mutational spectra in SD sequences are a 27.1% increase in C>G mutations, a 15.3% increase in C>A mutations and a 10.5% increase in A>C mutations. C>G mutations are associated with double-strand breaks in humans and some other apes^42,43^. This effect becomes more pronounced (+40.4%) in our candidate IGC regions consistent with previous observations showing increases in C>G mutations in regions of non-crossover gene conversion and double-strand breaks^43–45^. However, the increase remains in SD regions without IGC (+20.0%) perhaps owing to extensive nonallelic homologous recombination associated with SDs or undetected IGC events^4,9^.

To further investigate the potential effect of GC-biased gene conversion (gBGC) on the mutational spectra in SDs, we measured the frequency of (A,T)>(G,C) mutations in SD regions with evidence of IGC to determine whether cytosine and guanine bases are being preferentially maintained as might be expected in regions undergoing gBGC. If we measure the frequency of (A,T)>(C,G) in windows with at least one haplotype showing evidence of IGC, then we observe that the frequency is 4.7% higher than in unique regions of the genome; notably, in SDs without IGC, this rate is reduced compared to that of unique sequence (−3.5%). Additionally, there is a 5.8% reduction in (G,C)>(A,T) bases consistent with IGC preferentially restoring CG bases that have mutated to AT bases through gBGC. These results indicate that gBGC between paralogous sequences may be a strong factor in shaping the mutational landscape of SDs. Although, the (A,T)>(C,G) frequency is comparable in SD regions not affected by IGC, the mutational landscape at large is still very distinct between SDs and unique parts of the genome. In PCA of the mutational spectra in SDs without IGC, the first principal component distinguishing the mutational spectrum of SDs and unique DNA captures a larger fraction of the variation (94.6%) than in the PCA including IGC sites (80.2%; Supplementary Fig. 20).

## Modelling of elevated SNV frequency

To model the combined effect of unique mutational properties, evolutionary age and sequence content on the frequency of SNVs, we developed a multivariable linear regression using copy number, SD identity, number of unique IGC events, GC content and TMRCA to predict the number of SNVs seen in a 10-kbp window. A linear model containing all pairwise interactions of these predictors was able to explain 10.5% of the variation in SNVs per 10 kbp (adjusted *R*^2^), whereas a model containing only the number of IGC events explained only 1.8% of the variation. We note that this measure of variance is related but not directly comparable to the finding that the elevation in the number of SNVs is reduced by 23% when excluding IGC regions. All of the random variables, including their pairwise interactions, were significant (*P* value < 0.05) predictors of SNVs per 10 kbp except the interaction of number of IGC events with GC content, copy number and TMRCA. The strongest single predictors were the number of unique IGC events and the divergence of the overlapping SD (Supplementary Table 14).

## Discussion

Since the first publications of the human genome^12,13^, the pattern of single-nucleotide variation in recently duplicated sequence has been difficult to ascertain, leading to errors^2,11^. Later, indirect approaches were used to infer true SNVs in SDs, but these were far from complete^40^. More often than not, large-scale sequencing efforts simply excluded such regions in an effort to prevent paralogous sequence variants from contaminating single-nucleotide polymorphism databases and leading to false genetic associations^8,23^. The use of phased genome assemblies as opposed to aligned sequence reads had the advantage of allowing us to establish 1:1 orthologous relationships as well as the ability to discern the effect of IGC while comparing the pattern of single-nucleotide variation for both duplicated and unique DNA within the same haplotypes. As a result, we identify over 1.99 million nonredundant SNVs in a gene-rich portion of the genome previously considered largely inaccessible.

SNV density is significantly elevated (60%) in duplicated DNA when compared to unique DNA consistent with suggestions from primate genome comparisons and more recent de novo mutation studies from long-read sequencing data^46–48^. Furthermore, an increased de novo mutation rate in SDs could support our observation of an elevated SNV density without the need for an increase in TMRCA. We estimate that at least 23% of this increase is due to the action of IGC between paralogous sequences that essentially diversify allelic copies through concerted evolution. IGC in SDs seems to be more pervasive in the human genome compared to earlier estimates^15,27^, which owing to mapping uncertainties or gaps could assay only a smaller subset of regions^15,27^. We estimate more than 32,000 candidate regions (including 799 protein-coding genes) with the average human haplotype showing 1,192 events when compared to the reference. The putative IGC events are also much larger (mean 6.26 kbp) than those of most previous reports^28,49^, with the top 10% of the size distribution >14.4 kbp in length. This has the net effect that entire genes are copied hundreds of kilobase pairs into a new genomic context when compared to the reference. The effect of such ‘repositioning events’ on gene regulation will be an interesting avenue of future research.

As for allelic gene conversion, our predicted nonallelic gene conversion events are abundant, cluster into larger regional hotspots and favour G and C mutations, although this last property is not restricted to IGC regions^45,50^. Although we classify these regions as putative IGC events, other mutational processes such as deletion followed by duplicative transposition could, in principle, generate the same signal creating large tracts of ‘repositioned’ DNA. It should also be stressed that our method simply relies on the discovery of a closer match within the reference; by definition, this limits the detection of IGC events to regions where the donor sequence is already present in the reference as opposed to an alternative. Moreover, we interrogated only regions where 1:1 synteny could be unambiguously established. As more of the genome is assessed in the context of a pangenome reference framework, we anticipate that the proportion of IGC will increase, especially as large-copy-number polymorphic SDs, centromeres and acrocentric DNA become fully sequence resolved^3^. Although we estimate 4.3 Mbp of IGC in SDs on average per human haplotype, we caution that this almost certainly represents a lower bound and should not yet be regarded as a rate until more of the genome is surveyed and studies are carried out in the context of parent–child trios to observe germline events.

One of the most notable features of duplicated DNA is its higher GC content. In this study, we show that there is a clear skew in the mutational spectrum of SNVs to maintain this property of SDs beyond expectations from unique DNA. This property and the unexpected Ti/Tv ratio cannot be explained by lower accuracy of the assembly of SD regions. We find a 27.1% increase in transversions that convert cytosine to guanine or the reverse across all triplet contexts. GC-rich DNA has long been regarded as hypermutable. For example, C>G mutations preferentially associate with double-strand breaks in humans and apes^42,43^ and GC-rich regions in yeast show about 2–5 times more mutations depending on sequence context compared to AT-rich DNA^41^. Notably, in human SD regions, we observe a paucity of CpG transition mutations, characteristically associated with spontaneous deamination of CpG dinucleotides and concomitant transitions^6^. The basis for this is unclear, but it may be partially explained by the recent observation that duplicated genes show a greater degree of hypomethylation when compared to their unique counterparts^10^. We propose that excess of guanosine and cytosine transversions is a direct consequence of GC-biased gene conversion^5^ driven by an excess of double-strand breaks that result from a high rate of nonallelic homologous recombination events and other break-induced replication mechanisms among paralogous sequences.

## Methods

### Defining unique and SD regions

To define regions of SD, we used the annotations available for T2T-CHM13 v1.1 (ref. ^10^), which include all nonallelic intrachromosomal and interchromosomal pairwise alignments >1 kbp and with >90% sequence identity that do not consist entirely of common repeats or satellite sequences^11^. To define unique regions, we found the coordinates in T2T-CHM13 that were not SDs, ancient SDs (<90% sequence identity), centromeres or satellite arrays^51^ and defined these areas to be the non-duplicated (unique) parts of the genome. For both SDs and unique regions, variants in tandem repeat elements as identified by Tandem Repeats Finder^52^ were excluded because many SNVs called in these regions are ultimately alignment artefacts. RepeatMasker v4.1.2 was used to annotate SNVs with additional repeat classes beyond SDs^53^.

### Copy number estimate validation

The goal of this analysis was to validate copy number from the assembled HPRC haplotypes compared to estimates from read-depth analysis of the same samples sequenced using Illumina whole-genome sequencing (WGS). Large, recently duplicated segments are prone to copy number variation and are also susceptible to collapse and misassembly owing to their repetitive nature. HPRC haplotypes were assembled using PacBio HiFi with hifiasm^3,54^ creating contiguous long-read assemblies. We selected 19 SD loci corresponding to genes that were known to be duplicated and copy number variable in the human species. We *k*-merized the 2 haplotype assemblies corresponding to each locus for each individual into *k*-mers of 31 base pairs in length. We then computed copy number estimates over each locus for the sum haplotype assemblies and calculated the difference based on Illumina WGS from the same sample. For both datasets, we derived these estimates using FastCN, an algorithm implementing whole-genome shotgun sequence detection^55^. When averaging across each region and comparing differences in assembly copy versus Illumina WGS copy estimate, we observe that 756 out of 893 tests were perfectly matched (*δ* = 0), suggesting that most of these assemblies correctly represent the underlying genomic sequence of the samples.

### Quality value estimations with Merqury

Estimates of the quality value of SD and unique regions were made using Merqury v1.1 and parental Illumina sequencing data^56^. We first used Meryl to create *k*-mer databases (with a *k*-mer length of 21) using the parental sequencing data following the instructions in the Merqury documentation. Then Merqury was run with default parameters (merqury.sh {k-mer meryl database} {paternal sequence} {maternal sequence}) to generate quality value estimates for the hifiasm assemblies.

### Haplotype integrity analysis using inter-SUNK approach

For the 35 HPRC assemblies with matched ultralong Oxford Nanopore Technologies (ONT) data, we applied GAVISUNK v1.0.0 as an orthogonal validation of HiFi assembly integrity^57^. In brief, candidate haplotype-specific singly unique nucleotide *k*-mers (SUNKs) of length 20 are determined from the HiFi assembly and compared to ONT reads phased with parental Illumina data. Inter-SUNK distances are required to be consistent between the assembly and ONT reads, and regions that can be spanned and tiled with consistent ONT reads are considered validated. ONT read dropouts do not necessarily correspond to misassembly—they are also caused by large regions devoid of haplotype-specific SUNKs from recent duplications, homozygosity or over-assembly of the region, as well as Poisson dropout of read coverage.

### Read-depth analysis using the HPRC unreliable callset

For the 94 assembled HPRC haplotypes, we downloaded the regions identified to have abnormal coverage form S3 (s3://human-pangenomics/submissions/e9ad8022-1b30-11ec-ab04-0a13c5208311–COVERAGE_ANALYSIS_Y1_GENBANK/FLAGGER/JAN_09_2022/FINAL_HIFI_BASED/FLAGGER_HIFI_ASM_SIMPLIFIED_BEDS/ALL/). We then intersected these regions with the callable SD regions in each assembly to determine the number of collapsed, falsely duplicated and low-coverage base pairs in each assembly. The unreliable regions were determined by the HPRC using Flagger v0.1 (https://github.com/mobinasri/flagger/)^3^.

### Whole-genome alignments and synteny definition

Whole-genome alignments were calculated against T2T-CHM13 v1.1 with a copy of GRCh38 chrY using minimap2 v2.24 (ref. ^58^) with the parameters -a -x asm20–secondary=no -s 25000 -K 8G. The alignments were further processed with rustybam v0.1.29 (ref. ^59^) using the subcommands trim-paf to remove redundant alignments in the query sequence and break-paf to split alignments on structural variants over 10 kbp. After these steps, the remaining alignments over 1 Mbp of continuously aligned sequence were defined to be syntenic. The software pipeline is available on GitHub at https://github.com/mrvollger/asm-to-reference-alignment/ (refs. ^58–67^).

### Estimating the diversity of SNVs in SDs and unique sequences

When enumerating the number of SNVs, we count all pairwise differences between the haplotypes and the reference, counting events observed in multiple haplotypes multiple times. Therefore, except when otherwise indicated, we are referring to the total number of pairwise differences rather than the total number of nonredundant SNVs (number of segregation sites). The software pipeline is available on GitHub at https://github.com/mrvollger/sd-divergence (refs. ^60–63,65,66,68^).

### Defining IGC events

Each query haplotype genome sequence was aligned to the reference genome (T2T-CHM13 v1.1) using minimap2 v2.24 (ref. ^58^) considering only those regions that align in a 1:1 fashion for >1 Mbp without any evidence of gaps or discontinuities greater than 10 kbp in size. This eliminates large forms of structural variation, including copy number variants or regions of large-scale inversion restricting the analysis to largely copy number invariant SD regions (about 120 Mbp) and flanking unique sequence. Once these syntenic alignments were defined, we carried out a second alignment fragmenting the 1:1 synteny blocks into 1-kbp windows (100-bp increments) and remapped back to T2T-CHM13 to identify each window’s single best alignment position. These second alignments were then compared to original syntenic ones and if they no longer overlapped, we considered them to be candidate IGC regions. Adjacent IGC windows were subsequently merged into larger intervals when windows continued to be mapped non-syntenically with respect to the original alignment. We then used the CIGAR string to identify the number of matching and mismatching bases at the ‘donor’ site and compared that to the number of matching and mismatching bases at the acceptor site determined by the syntenic alignment. A donor sequence is, thus, defined as a segment in T2T-CHM13 that now maps with higher sequence identity to a new location in the human haplotype (alignment method 2) and the acceptor sequence is the segment in T2T-CHM13 that has an orthologous mapping to the same region in the human haplotype (alignment method 1). As such, there is dependence on both the reference genome and the haplotype being compared. The software pipeline is available on GitHub at https://github.com/mrvollger/asm-to-reference-alignment/ (refs. ^58–67^).

### Assigning confidence to IGC events

To assign confidence measures to our IGC events, we adapted a previously described method^69^ to calculate a *P* value for every one of our candidate IGC calls. Our method uses a cumulative binomial distribution constructed from the number of SNVs supporting the IGC event and the total number of informative sites between two paralogues to assign a one-sided *P* value to each event. Specifically:

$$P(X\le k)=B(k,n,p)$$

in which *B* is the binomial cumulative distribution, *n* is the number of informative sites between paralogues, *k* is the number of informative sites that agree with the non-converted sequence (acceptor site), and *p* is the probability that at an informative site the base matches the acceptor sequence. We assume *p* to be 0.5 reflecting that a supporting base change can come from one of two sources: the donor or acceptor paralogue. With these assumptions, our binomial model reports the probability that we observe *k* or fewer sites that support the acceptor site (that is, no IGC) at random given the data, giving us a one-sided *P* value for each IGC event. No adjustments were made for multiple comparisons.

### Testing for IGC in unique regions

To test the specificity of our method, we applied it to an equivalent total of unique sequence (125 Mbp) on each haplotype, which we expected to show no or low levels of IGC. On average, we identify only 33.5 IGC events affecting 38.2 kbp of sequence per haplotype. If we restrict this to high-confidence IGC events, we see only 5.93 events on average affecting 7.29 kbp. This implies that our method is detecting IGC above background in SDs and that the frequency of IGC in SDs is more than 50 times higher in the high-confidence callsets (31,910 versus 605).

### Additional genome assemblies

We assembled HG00514, NA12878 and HG03125 using HiFi long-read data and hifiasm v0.15.2 with parental Illumina data^54^. Using HiFi long-read data and hifiasm v0.15.2 we also assembled the genome of the now-deceased chimpanzee Clint (sample S006007). The assembly is locally phased as trio-binning and HiC data were unavailable. Data are available on the National Center for Biotechnology Information (NCBI) Sequence Read Archive (SRA) under the BioProjects PRJNA551670 (ref. ^4^), PRJNA540705 (ref. ^70^), PRJEB36100 (ref. ^4^) and PRJNA659034 (ref. ^47^). These assemblies are made available on Zenodo (10.5281/zenodo.6792653)^71^.

### Determining the composition of triplet mutations in SD and unique sequences

The mutational spectra for unique and SD regions from each individual were computed using mutyper on the basis of derived SNVs polarized against the chimpanzee genome assembly described above^72–74^. These spectra were normalized to the triplet content of the respective unique or SD regions by dividing the count of each triplet mutation type by the total count of each triplet context in the ancestral region and normalizing the number of counts in SD and unique sequences to be the same. For PCA, the data were further normalized using the centred log-ratio transformation, which is commonly used for compositional measurements^75^. The code is available on GitHub at https://github.com/mrvollger/mutyper_workflow/ (refs. ^61–63,65,72,76^).

### Estimation of TMRCA

To estimate TMRCA for a locus of interest, we focus on orthologous sequences (10-kbp windows) identified in synteny among human and chimpanzee haplotypes. Under an assumption of infinite sites, the number of mutations ${x}_{i}$ between a human sequence and its most recent common ancestor is Poisson distributed with a mean of $\mu \times T$, in which $\mu$ is the mutation rate scaled with respect to the substitutions between human and chimpanzee lineages, and *T* is the TMRCA. That is, $T={\sum }_{i=1}^{n}{x}_{i}/n\mu$, in which *n* is the number of human haplotypes. To convert TMRCA to time in years, we assume six million years of divergence between human and chimpanzee lineages. We note that the TMRCA estimates reported in the present study account for mutation variation across loci (that is, if the mutation rate is elevated for a locus, the effect would be accounted for). Thus, for each individual locus, an independent mutation (not uniform) rate is applied depending on the observed pattern of mutations compared to the chimpanzee outgroup.

### Reporting summary

Further information on research design is available in the Nature Portfolio Reporting Summary linked to this article.

## Online content

Any methods, additional references, Nature Portfolio reporting summaries, source data, extended data, supplementary information, acknowledgements, peer review information; details of author contributions and competing interests; and statements of data and code availability are available at 10.1038/s41586-023-05895-y.

### Supplementary information

Supplementary Information This file contains Supplementary Figs. 1–20, Notes and References.

Reporting Summary

Supplementary Tables This file contains Supplementary Tables 1–14.

## Figures and Tables

**Extended Data Fig. 7:**
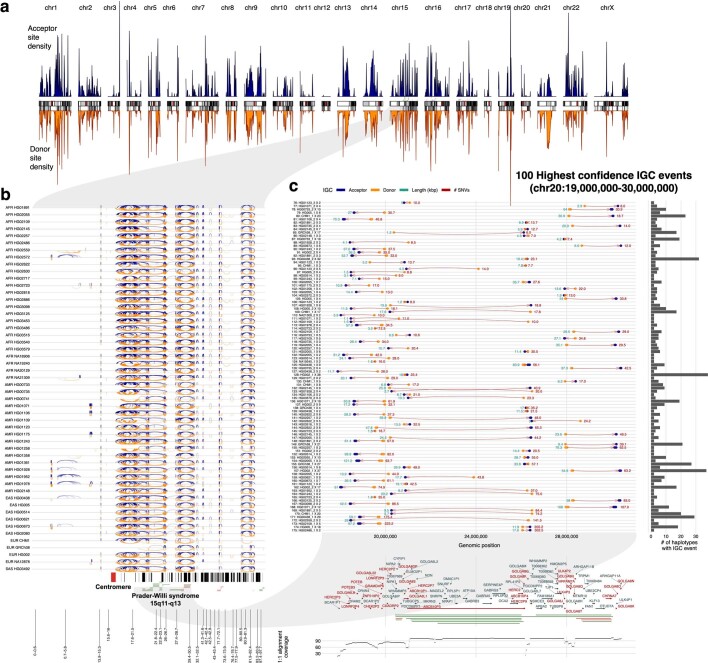
IGC hotspots. **a)** Density of IGC acceptor (top, blue) and donor (bottom, orange) sites across the “SD genome”. The SD genome consists of all main SD regions (>50 kbp) minus the intervening unique sequences. **b)** All intrachromosomal IGC events from 102 human haplotypes analyzed for chromosome 15. Arcs drawn in blue (top) have the acceptor site on the left-hand side and the donor site on the right. Arcs drawn in orange (bottom) are arranged oppositely. Protein-coding genes are drawn as vertical black lines above the ideogram, and large duplication (blue) and deletion (red) events associated with human diseases are drawn as horizontal lines just above the ideogram. **c)** Zoom of the 100 highest confidence (lowest p-value) IGC events identified on chromosome 15 between 17 and 31 Mbp. Genes that are intersected by IGC events are highlighted in red.

## Data Availability

PacBio HiFi and ONT data have been deposited into NCBI SRA under the following BioProject IDs: PRJNA850430, PRJNA731524, PRJNA551670, PRJNA540705 and PRJEB36100. PacBio HiFi data for CHM1 are available under the following SRA accessions: SRX10759865 and SRX10759866. Sequencing data for Clint PTR are available on NCBI SRA under the BioProject PRJNA659034. The T2T-CHM13 v1.1 assembly can be found on NCBI (GCA_009914755.3). Cell lines obtained from the NIGMS Human Genetic Cell Repository at the Coriell Institute for Medical Research are listed in Supplementary Table 1. Assemblies of HPRC samples are available on NCBI under the BioProject PRJNA730822. All additional assemblies used in this work (Clint PTR, CHM1, HG00514, NA12878 and HG03125), variant calls, assembly alignments, and other annotation data used in analysis are available on Zenodo (10.5281/zenodo.6792653)^71^.

## Code availability

The software pipeline for aligning assemblies and calling IGC is available on GitHub (https://github.com/mrvollger/asm-to-reference-alignmentv0.1) and Zenodo (https://zenodo.org/record/7653446)^67^. Code for analysing variants called against T2T-CHM13 v1.1 is available on GitHub (https://github.com/mrvollger/sd-divergencev0.1 and Zenodo (https://zenodo.org/record/7653464)^68^. The software pipeline for analysing the triple context of SNVs is available on GitHub (https://github.com/mrvollger/mutyper_workflowv0.1) and Zenodo (https://zenodo.org/record/7653472)^76^. Scripts for figure and table generation are available on GitHub (https://github.com/mrvollger/sd-divergence-and-igc-figuresv0.1) and Zenodo (https://zenodo.org/record/7653486)^77^. GAVISUNK is available on GitHub (https://github.com/pdishuck/GAVISUNK) and Zenodo (https://zenodo.org/record/7655335)^57^.
