# Supplementary material for: Increased mutation and gene conversion within human segmental duplications
Source: Nature. 2023 May 10;617(7960):325–34. doi: 10.1038/s41586-023-05895-y (PMC10172114; doi:10.1038/s41586-023-05895-y)
Supplement: Supplementary file 1 — This file contains Supplementary Figs. 1–20, Notes and References. [file 41586_2023_5895_MOESM1_ESM.pdf]

nature portfolio

https://doi.org/10.1038/s41586-023-05895-y

Supplementary information

Increased mutation and gene conversion within human segmental duplications

In the format provided by the authors and unedited

Supplemental methods and information for:

Increased mutation and gene conversion within human segmental duplications

Table of Contents

Supplementary Figures 2
Supplementary Notes 22
Online Data 26
Supplementary References 27

Supplementary Figures

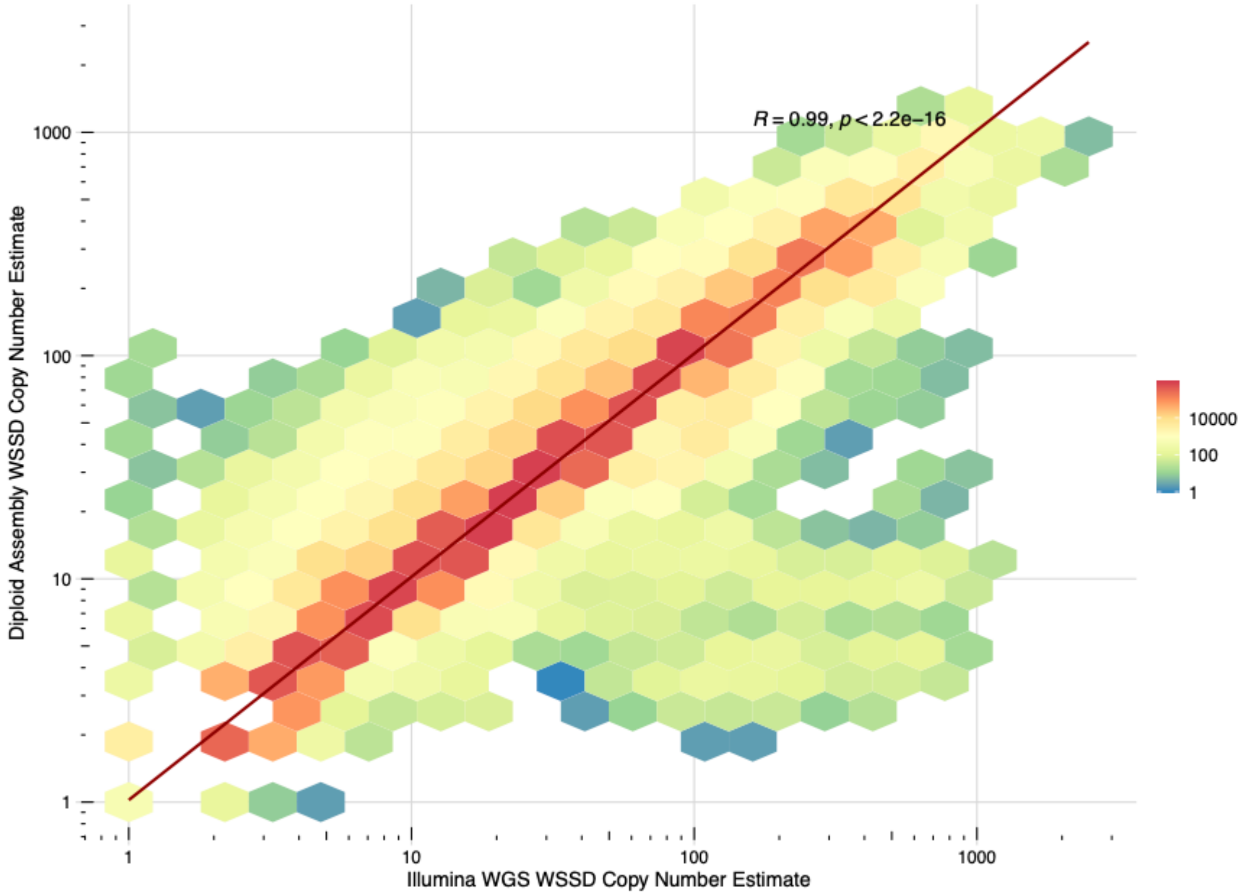

Figure S1. Genome-wide copy number estimate correlation. Shown is the correlation between each diploid assembly copy number estimate and the corresponding whole-genome shotgun sequence detection (WSSD) copy number estimate from Illumina libraries generated for the 1000 Genomes Project [1]. Data is included for all samples across all SD regions used in the analysis. The text indicates the value of the Pearson's correlation coefficient and the p-value from a two sided t-test without adjustment for multiple comparisons.

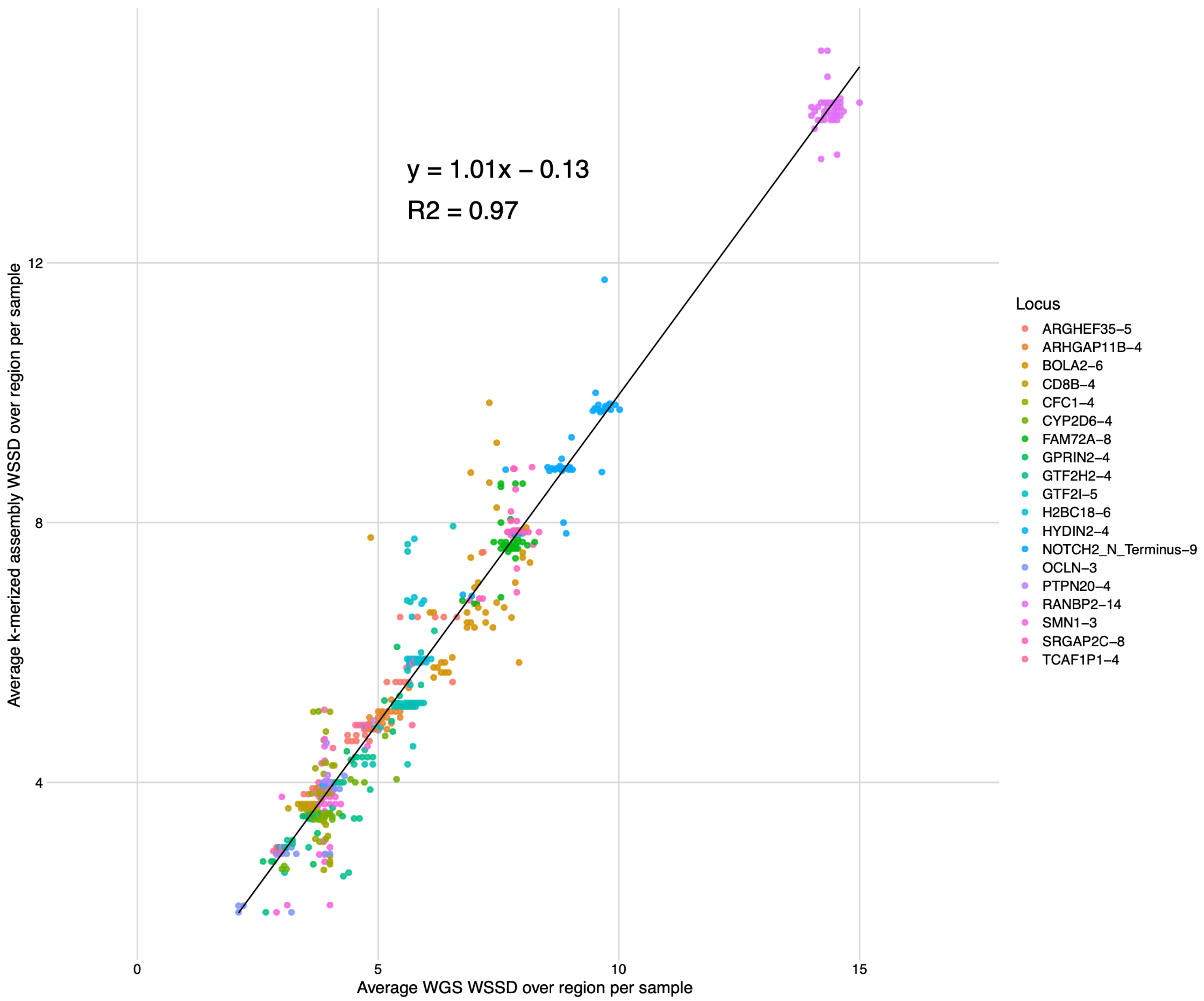

Figure S2. Copy number estimate correlation. Here, we illustrate the correlation between each sample's diplotype assembly copy number estimate and the corresponding WSSD copy number estimates from libraries generated in the 1000 Genomes Project for 19 selected SD loci. We observe a strong correlation between the two estimates (r2 = 0.97), indicating that, by copy number, the assemblies generated by the HPRC support native genomic copy number in these SD regions.

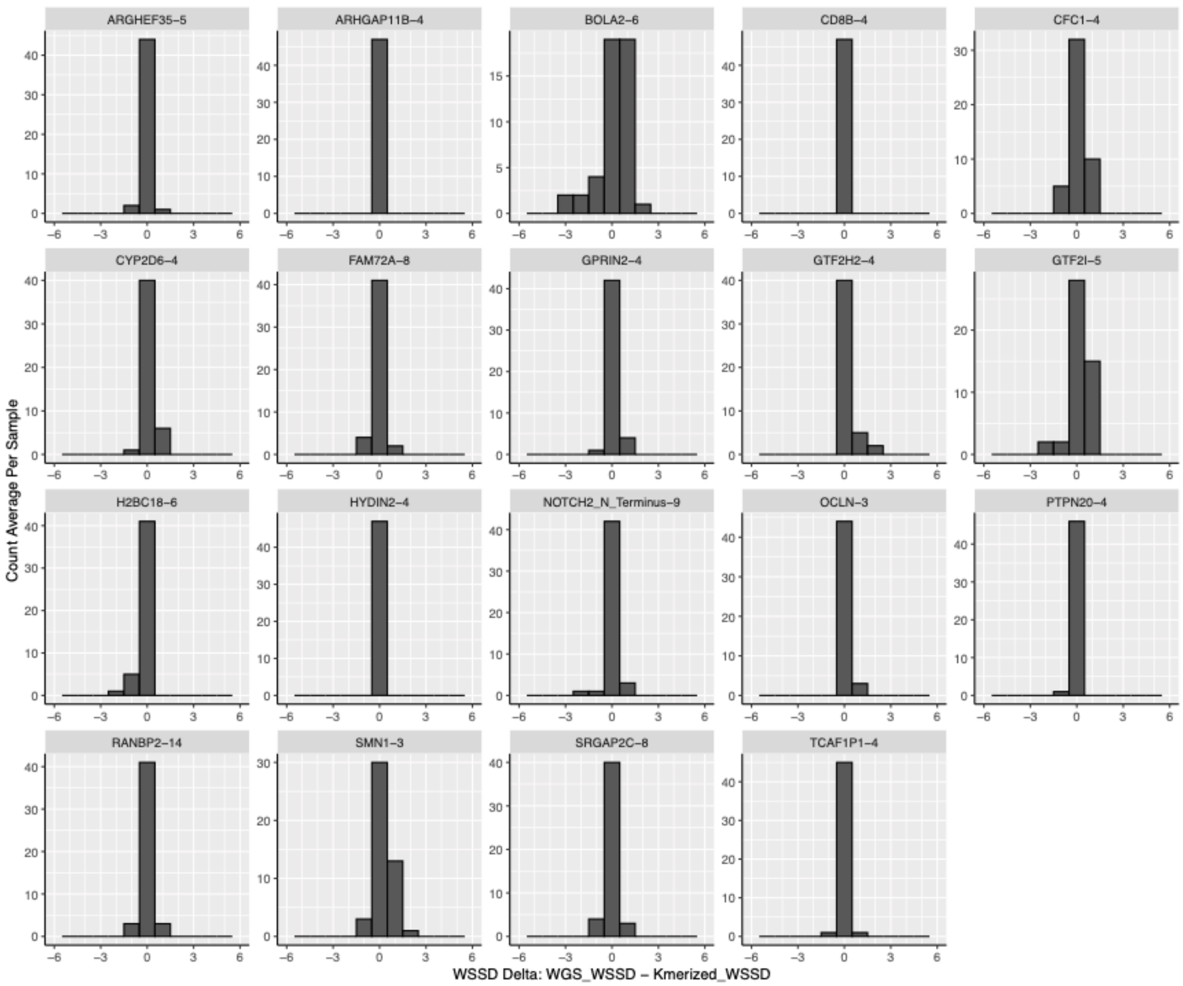

Figure S3. Average copy number estimate comparison. We estimated the copy number of 19 human SDs across 47 samples (94 haplotypes) using either k-mers aggregated from both assembly haplotypes and orthogonal Illumina data developed by the 1000 Genomes Project. Here, we illustrate the average copy number estimate differences over these 19 regions between the two methods. Titles of each histogram correspond to the gene models residing within and commonly associate with the particular human-specific duplication selected. Each number trailing the gene models are median copy number estimates for these regions across all 47 samples, estimated by diplotype assembly copy number. For the majority of tests, 756 of 893, copy number estimates are identical, resulting in a delta of zero. Diverging deltas most commonly occur in the largest and most highly identical regions but may also diverge due to region size or lower identity duplications.

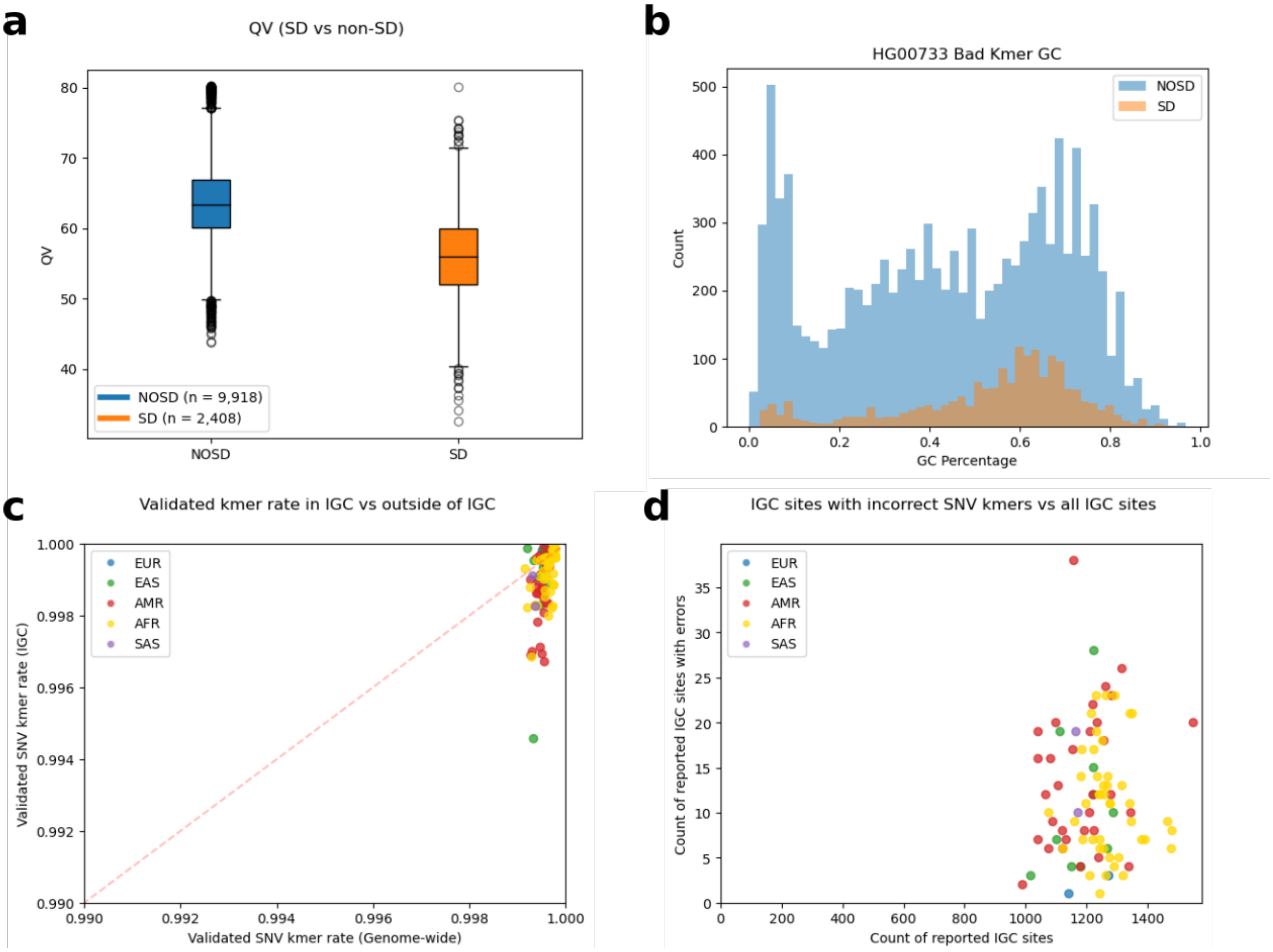

Figure S4. Assembly QV (quality value) in unique and SD regions and genome-wide error detection between IGC and non-IGC. a) QV distributions for 5 Mbp of syntenically aligned SD (n=2,408) and non-SD (NOSD, n=9,918) sequences across 45 samples from the HPRC-assembled genomes with Illumina sequencing data. The boxes indicate the range between the first and third quartiles, with the middle line indicating the median. The whiskers show the minimum and maximum value of the data that is within 1.5 times the interquartile range extending from the first and third quartiles, respectively. **b)** GC content of k-mers found in the assemblies but not in the Illumina libraries as identified by Merqury for HG00733. Median GC content of k-mers is higher (0.59 vs. 0.48) in k-mers identified in assemblies of SD sequence. **c)** Using discordant k-mers as a proxy of error, we estimated the percent of validated k-mers for regions classified as IGC versus the rest of the genome for 90 haplotypes where Illumina WGS data were available for HPRC assemblies. Both categories show high validation rates in line with their QV estimates. **d)** Comparing the number of IGC sites, which contain at least one assembly error (y-axis) as evaluated by Merqury, to the number of IGC sites, which do not contain any errors (x-axis), in SNVs reveals that the errors seen in the assemblies are localized to only a few IGC events. Additionally, the average number of SNVs that are incorrect in IGC sites with at least one error is 2.45, supporting the notion that these errors are clustered and infrequent. **Note:** For the calculation of QV, many syntenic SD and non-SD regions did not extend for 5 Mbp uninterrupted. In these cases, we concatenated multiple regions until 5 Mbp of sequence was identified in order to create comparable matched lengths between the two categories. As part of this analysis, we added 100 N’s where gaps existed between sequences so as to not create new k-mers not present in the assembly by joining contig sequences.

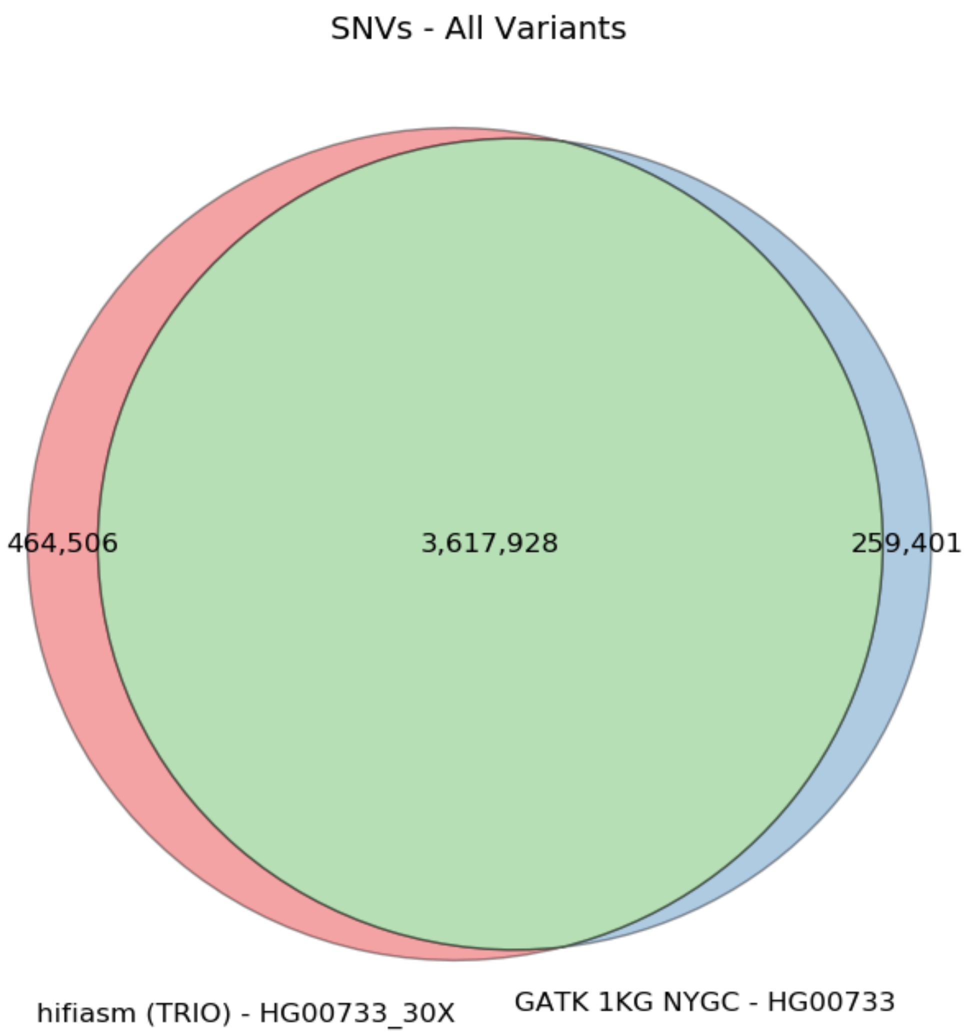

Figure S5. HiFi versus Illumina SNV callset comparison. Overlap of SNVs called from hifiasm assembly with PAV compared to the GATK calls generated by the New York Genome Center (NYGC) on high-coverage Illumina WGS for the same sample HG00733.

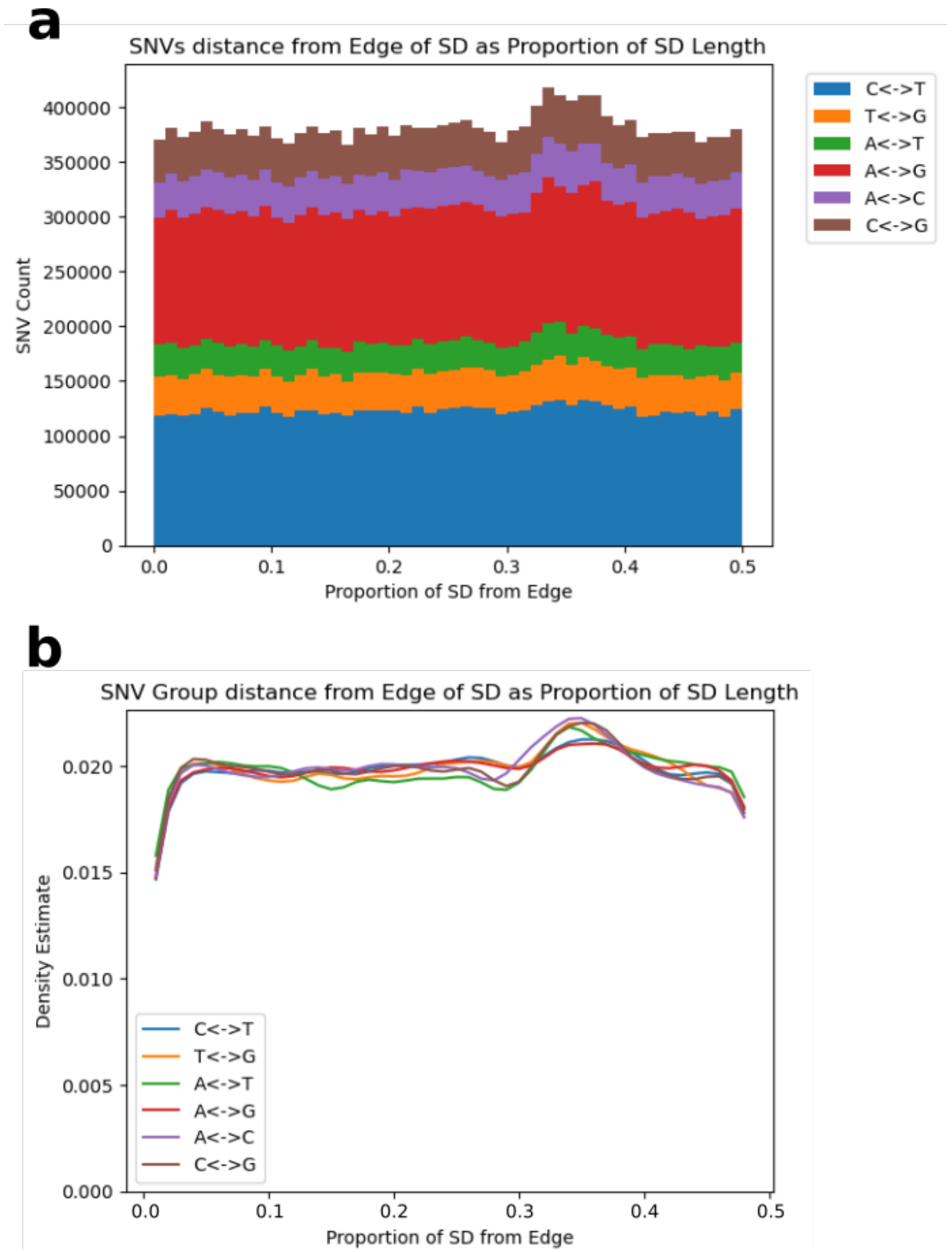

Figure S6. Mutational properties of SNVs as a function of positioning within SDs. a) Raw count of SNVs contained in SDs aggregated across all samples as a function of their distance from the edge of the SD sequence. 0.0 indicates an SNV on the edge of an SD while 0.5 represents an SNV right in the middle of the SD block. There may be a slight increase in SNV count in the internal sequence range (from 0.3 to 0.4 length to the edge); however, this is in the range of variation which could be attributed to noise. **b)** Density estimate of SNVs contained in SDs aggregated across all samples as a function of their distance from the edge of the SD sequence. 0.0 indicates an SNV on the edge of an SD while 0.5 represents an SNV right in the middle of the SD block. These relative distributions show that each type of variant is evenly distributed across SD space.

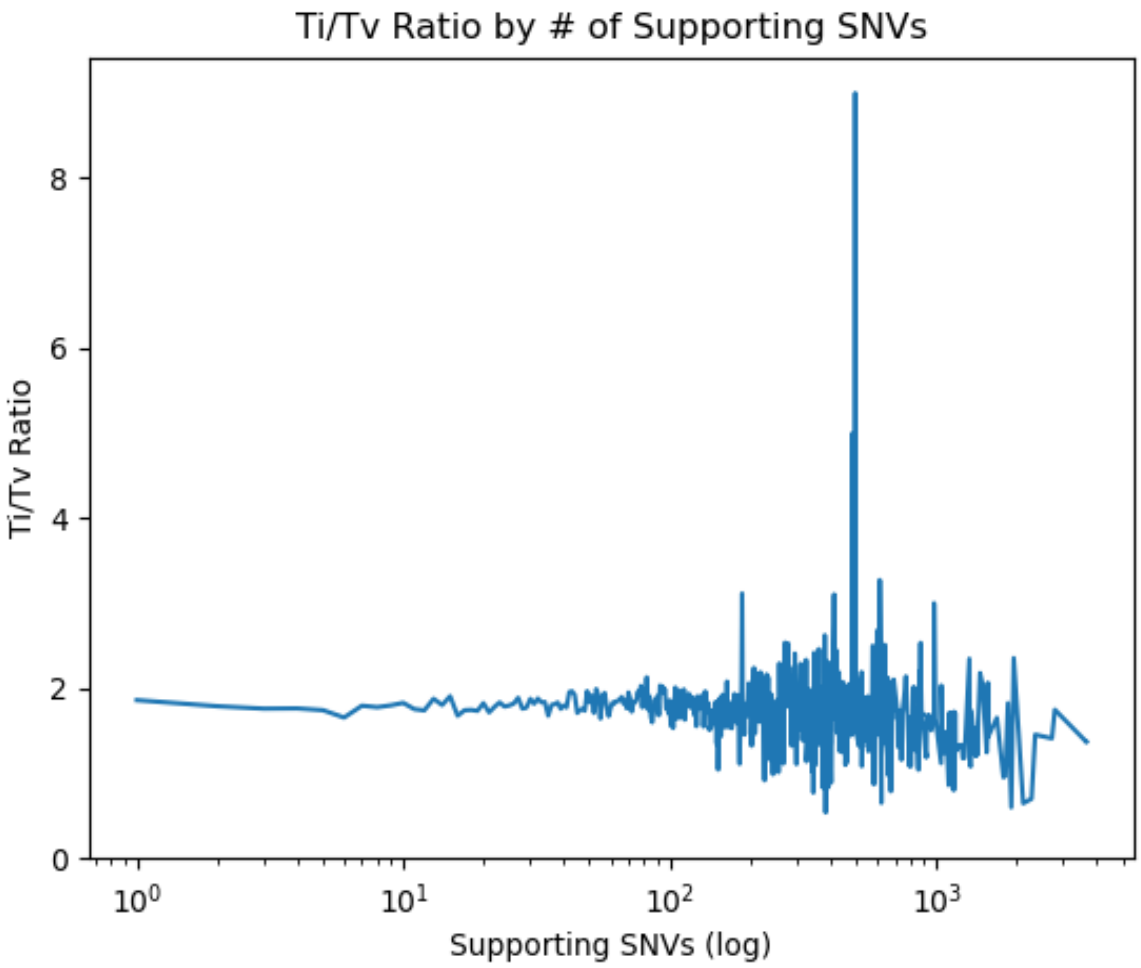

Figure S7. Ti/Tv ratio of IGC events by number of supporting SNVs. Ti/Tv remains consistent, especially below 100 supporting SNVs, with a mean of 1.83 and standard deviation of 0.09. Above that threshold, there is more variation likely due to less genomic space being represented in these bins.

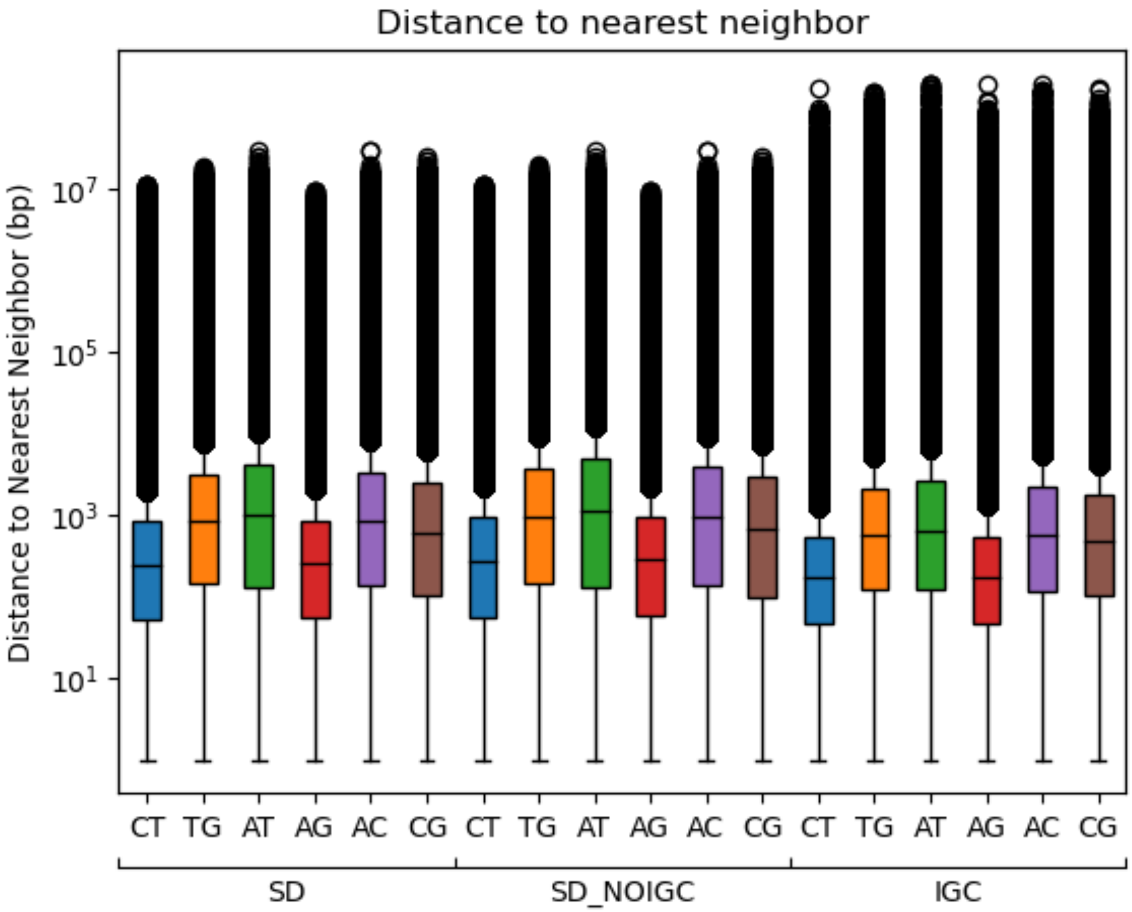

Figure S8. Distance between SNVs and their nearest neighbor of the same type. The distance between SNVs and their nearest neighbor of the same type shows a consistent pattern between SDs (n=2,025,990 independent SNVs), SDs without IGC (SD_NOIGC, n=1,213,196 independent SNVs), and IGC loci (n=842,133 independent SNVs). The boxes indicate the range between the first and third quartiles, with the middle line indicating the median. The whiskers show the minimum and maximum value of the data that is within 1.5 times the interquartile range extending from the first and third quartiles, respectively.

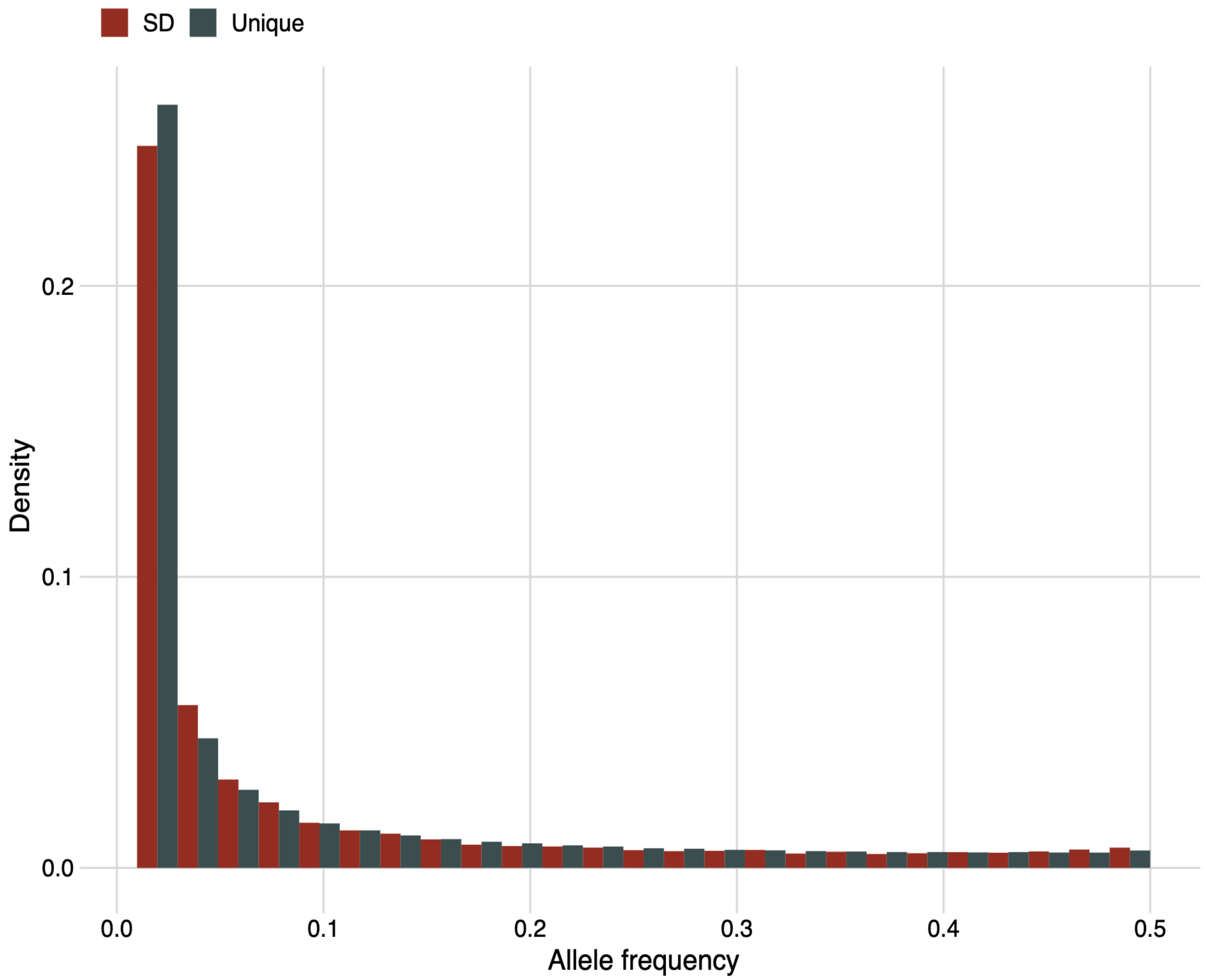

Figure S9. Allele frequency distribution. The histogram compares the SNV allele frequency distribution in unique (gray) versus SD regions (red) based on our analysis of 102 human haplotypes and SNVs defined by rustybam + dipcall.

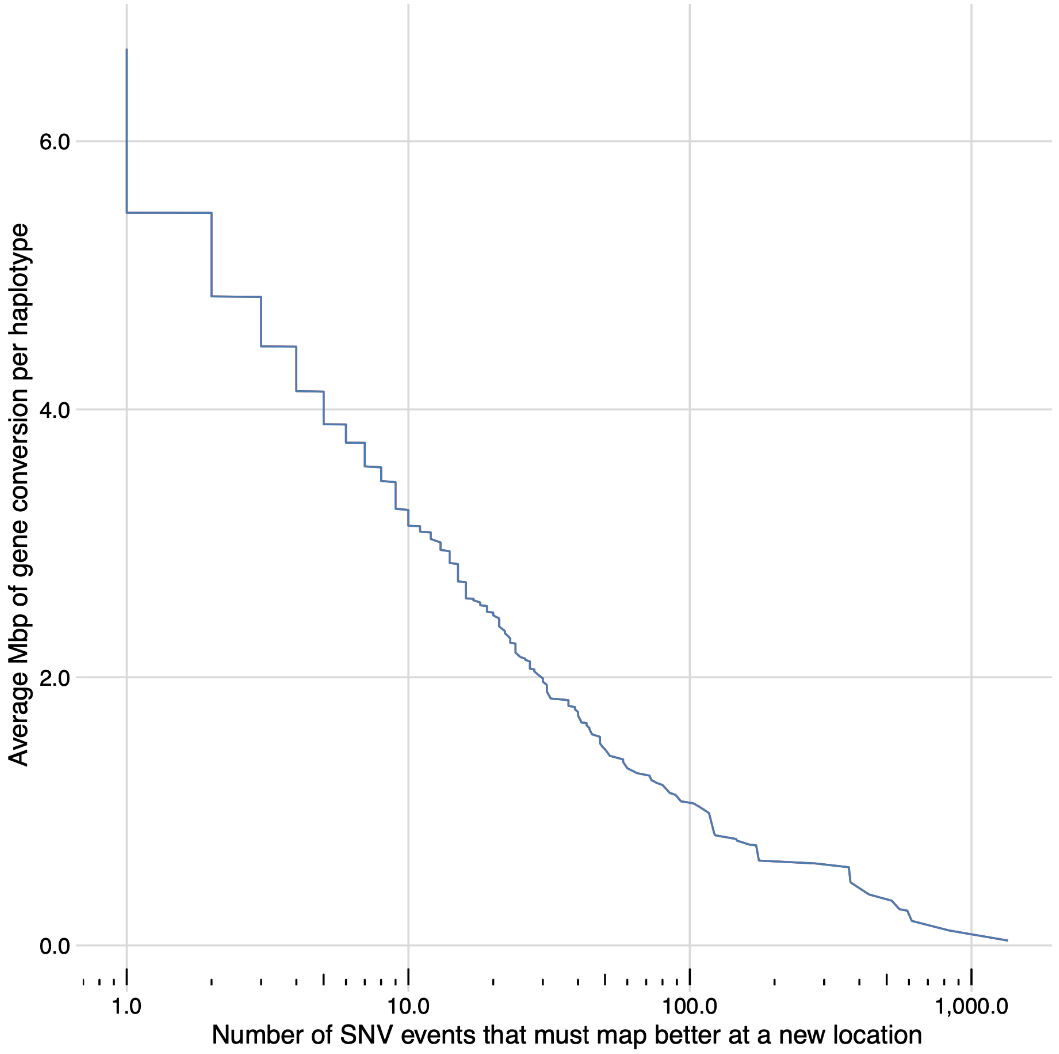

Figure S10. IGC in CHM1. The plot shows the average length (Mbp) of predicted IGC as a function of the number of SNVs supporting that length. For example, approximately 3.9 Mbp of IGC is predicted when requiring five SNV differences supporting that alignment to the new location. The CHM1 reference represents a single haplotype derived from a hydatidiform mole and thus provides a control for our analysis of diploid genomes where switch error and phasing issues could possibly confound the results.

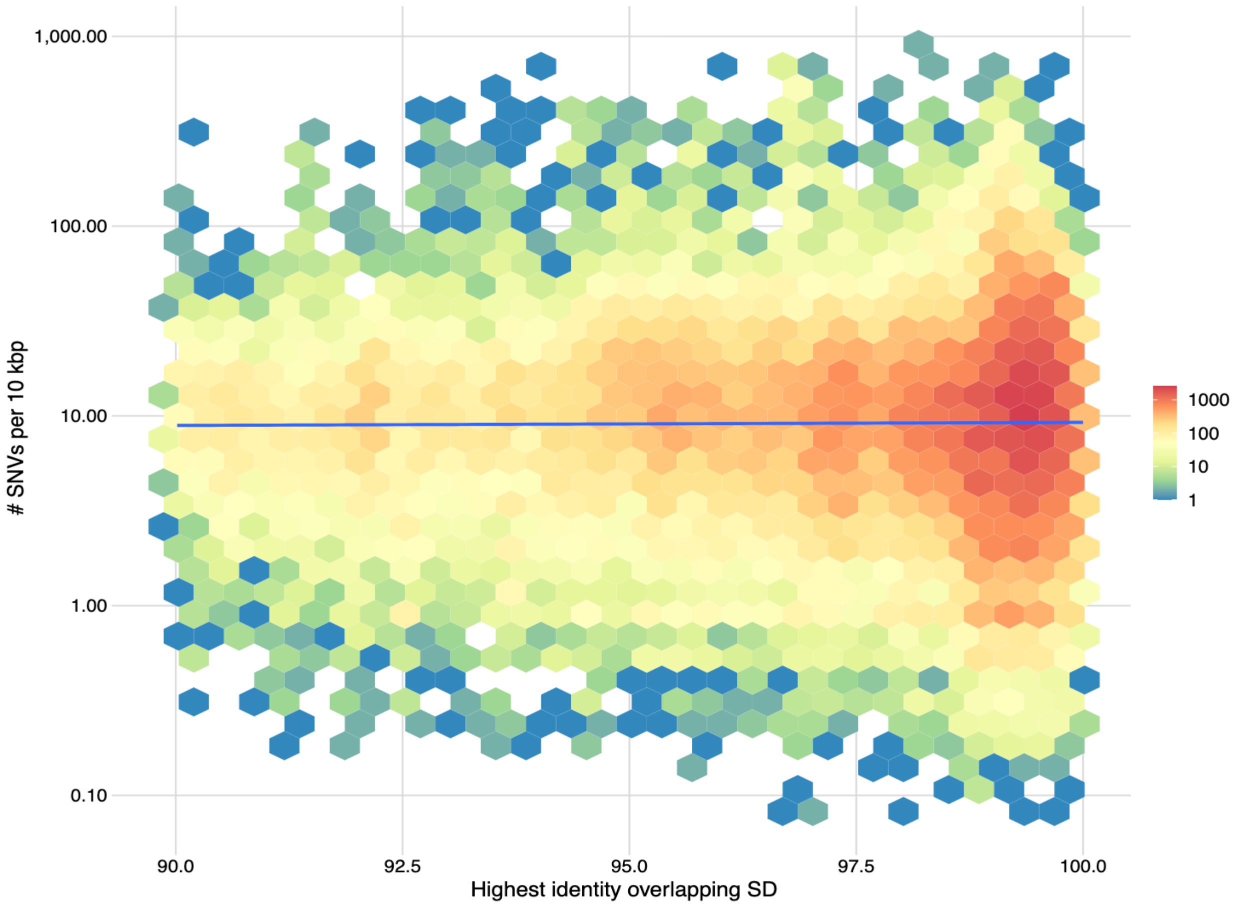

Figure S11. The average number of SNVs in 10 kbp windows across all the haplotypes as a function of highest identity overlapping SD. Colors reflect the number of counts within a given hex, and the regression line is shown in blue, indicating that SD identity has little to no effect on the number of SNVs.

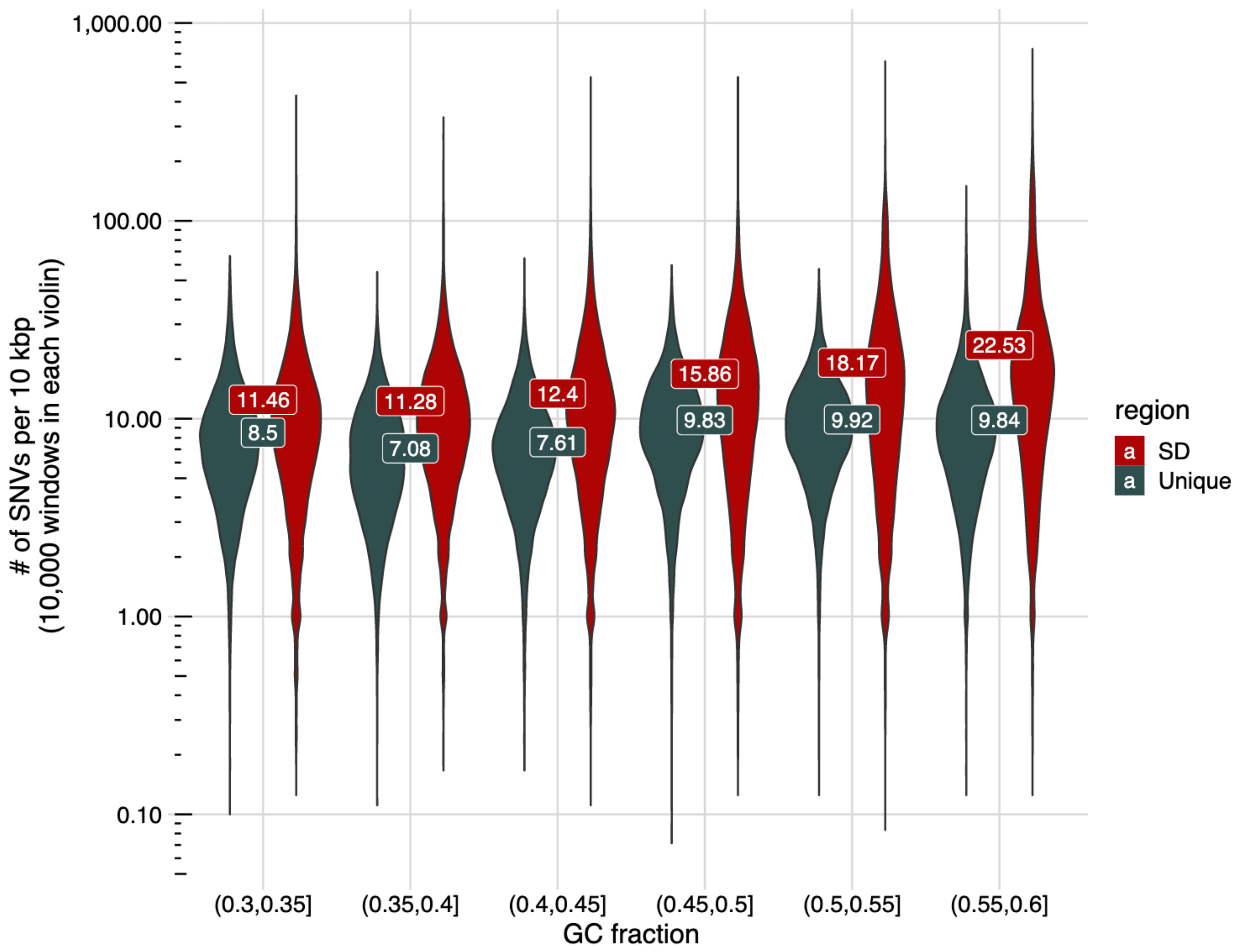

Figure S12. Average number of SNVs per 10 kbp across varying GC fractions. Shown is the average number of SNVs per 10 kbp window when considering different GC fractions. To create the violins, we randomly sampled 10,000 10 kbp windows from each GC fraction in both SD and unique regions. GC fractions that did not contain at least 10,000 independent 10 kbp windows for SD and unique sequences (i.e., GC < 0.3 or GC > 0.60) were excluded from the analysis so that all distributions could be made using the same number of observations.

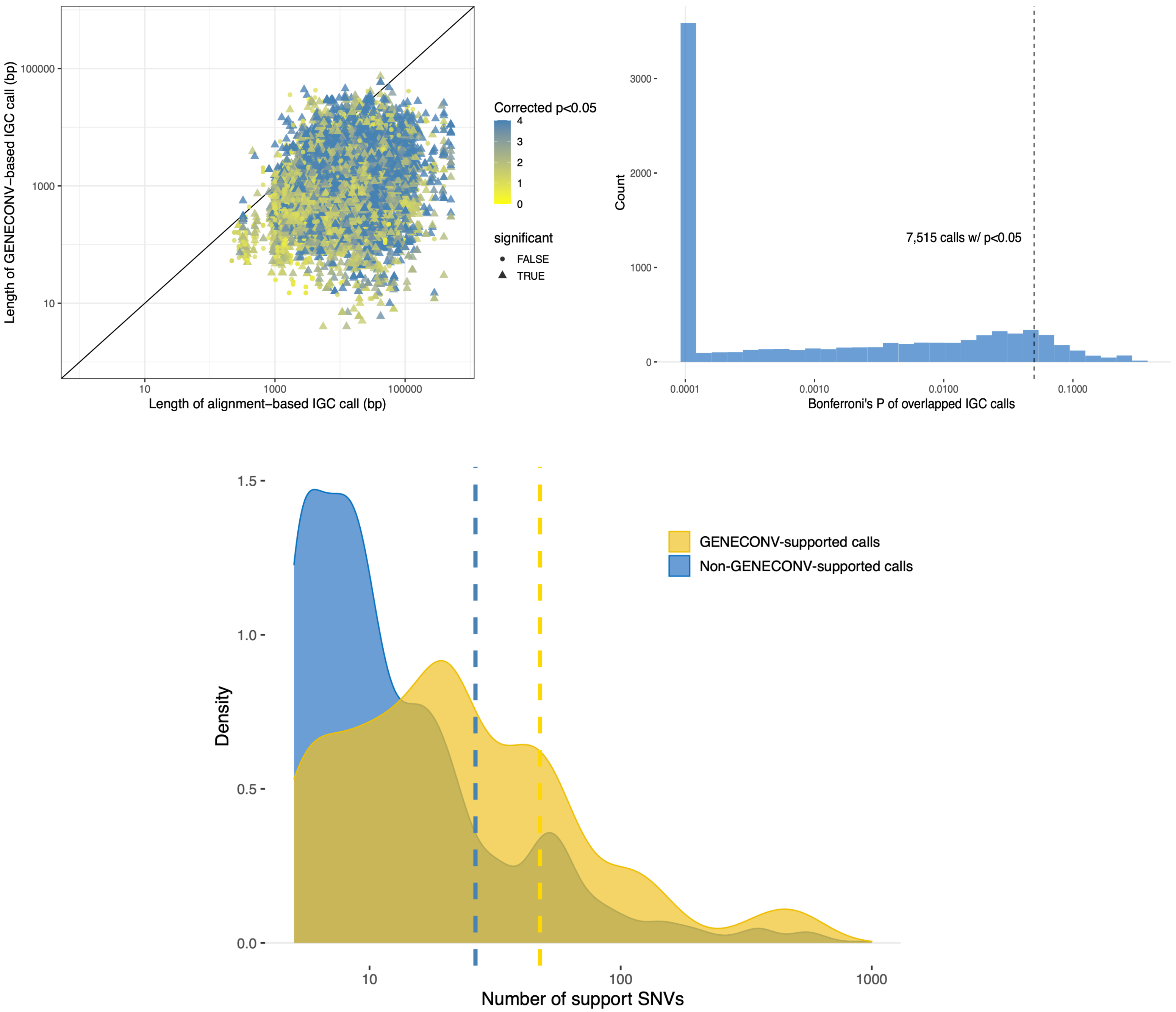

Figure S13. Alignment-based IGC calls versus GENECONV.
top left) Comparison of the length distributions of overlap IGC calls between the alignment-based and GENECONV-based methods [2,3]. Colors indicate the Bonferroni-corrected p-values reported by GENECONV on logarithmic scale (log10), and triangles and circles represent significant (Bonferroni-corrected $p < 0.05$) and insignificant calls [2,3]. **top right)** Histogram of Bonferroni-corrected p-values from GENECONV for the 7,515 nonredundant IGC calls detected by both methods. **bottom)** Distributions of support SNVs for alignment-based IGC calls overlapping and not overlapping GENECONV-based calls. Blue and yellow dashed lines represent the means of support SNVs in alignment-based only calls (mean support SNVs: 33.9) and those with GENECONV supports (mean support SNVs: 56.7).

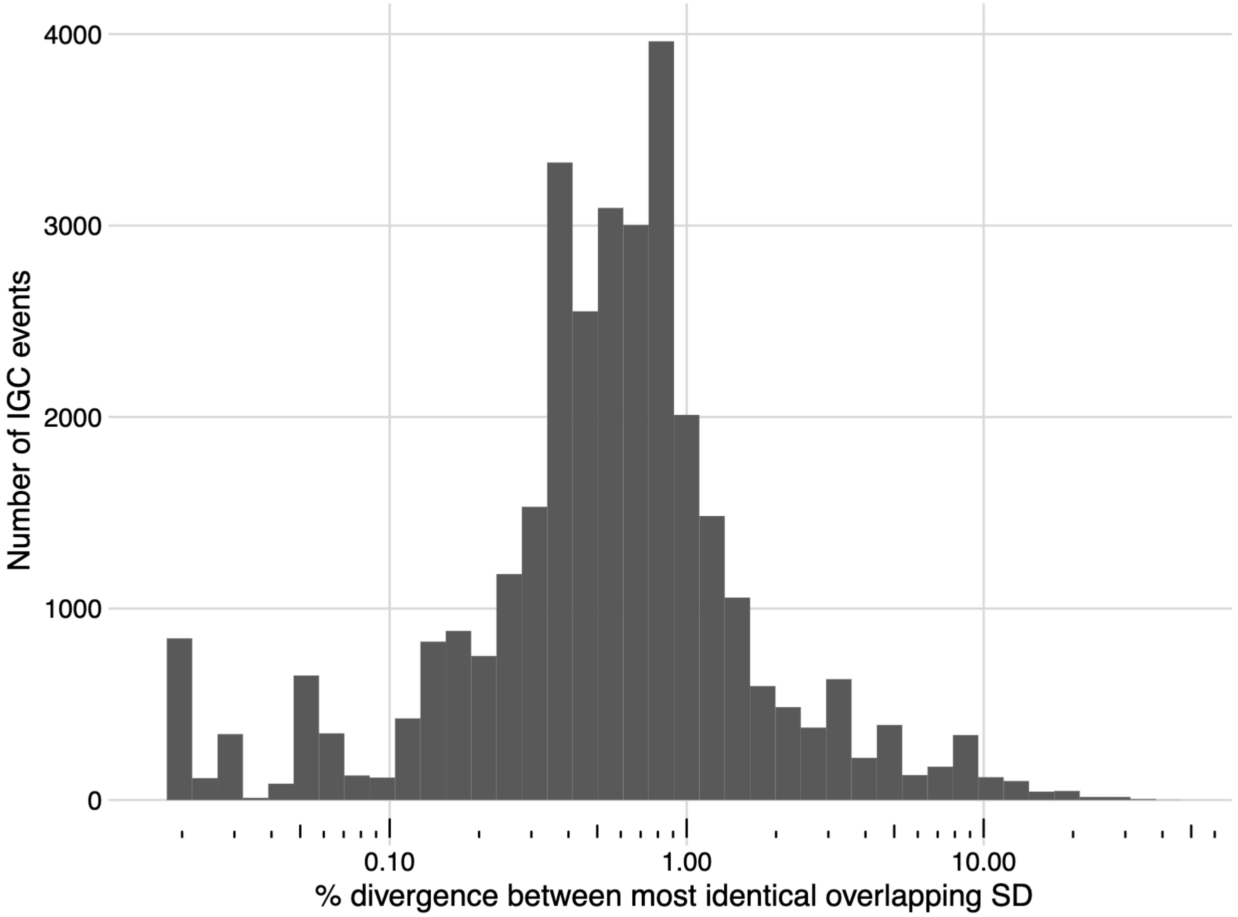

Figure S14. Number of IGC events as a function of the percent divergence between SDs. Shown is the percent divergence of the most highly identical overlapping SD for each putative IGC event. As expected, most events happen when there is high sequence identity with only 325 events happening in locations with >10% sequence divergence.

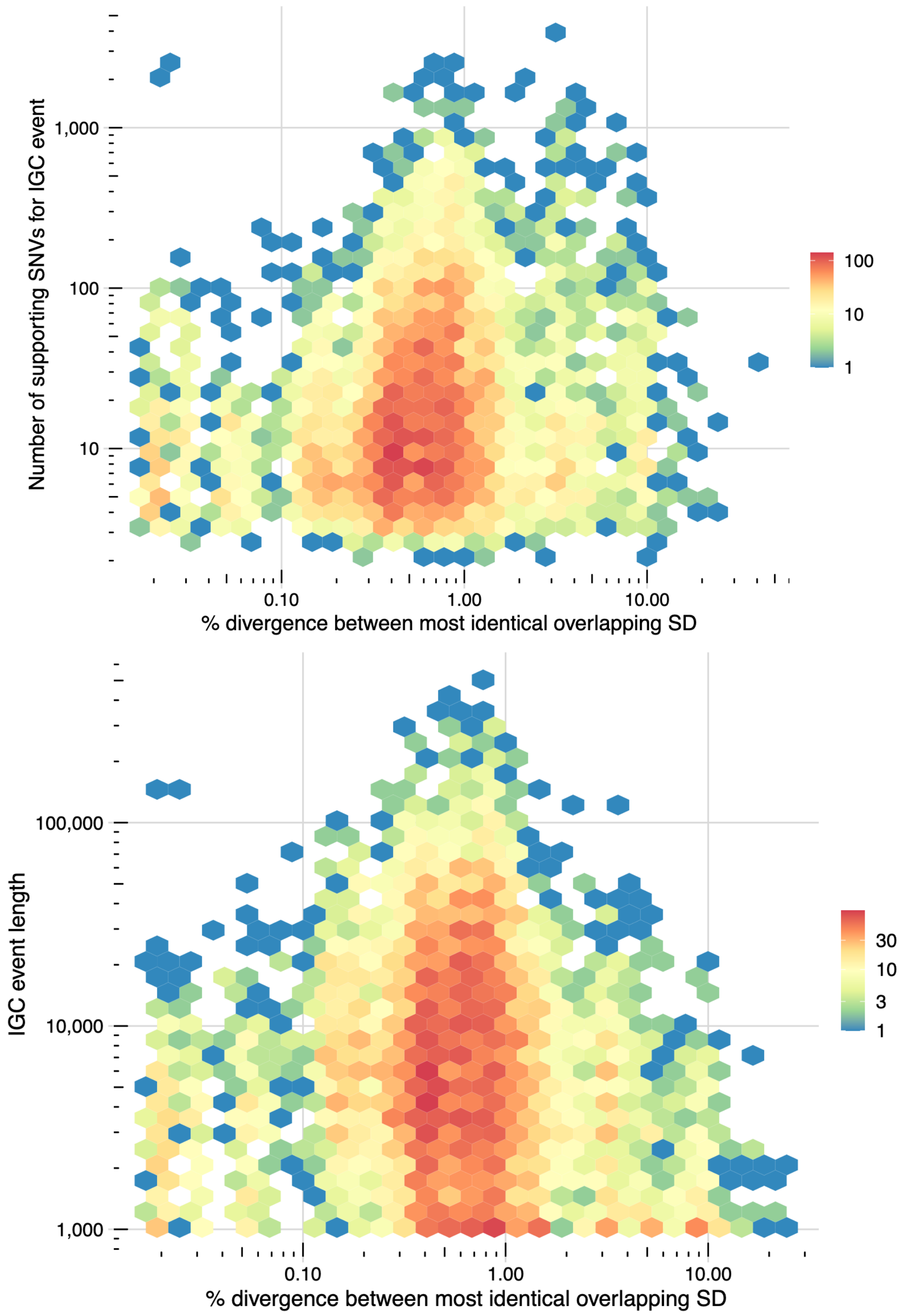

Figure S15. IGC as a function of paralog divergence.
top) Comparison of the number of supporting SNVs for an IGC event and the percent divergence of the most identical overlapping SD. **bottom)** Comparison of the length of IGC events and the percent divergence of the most identical overlapping SD.

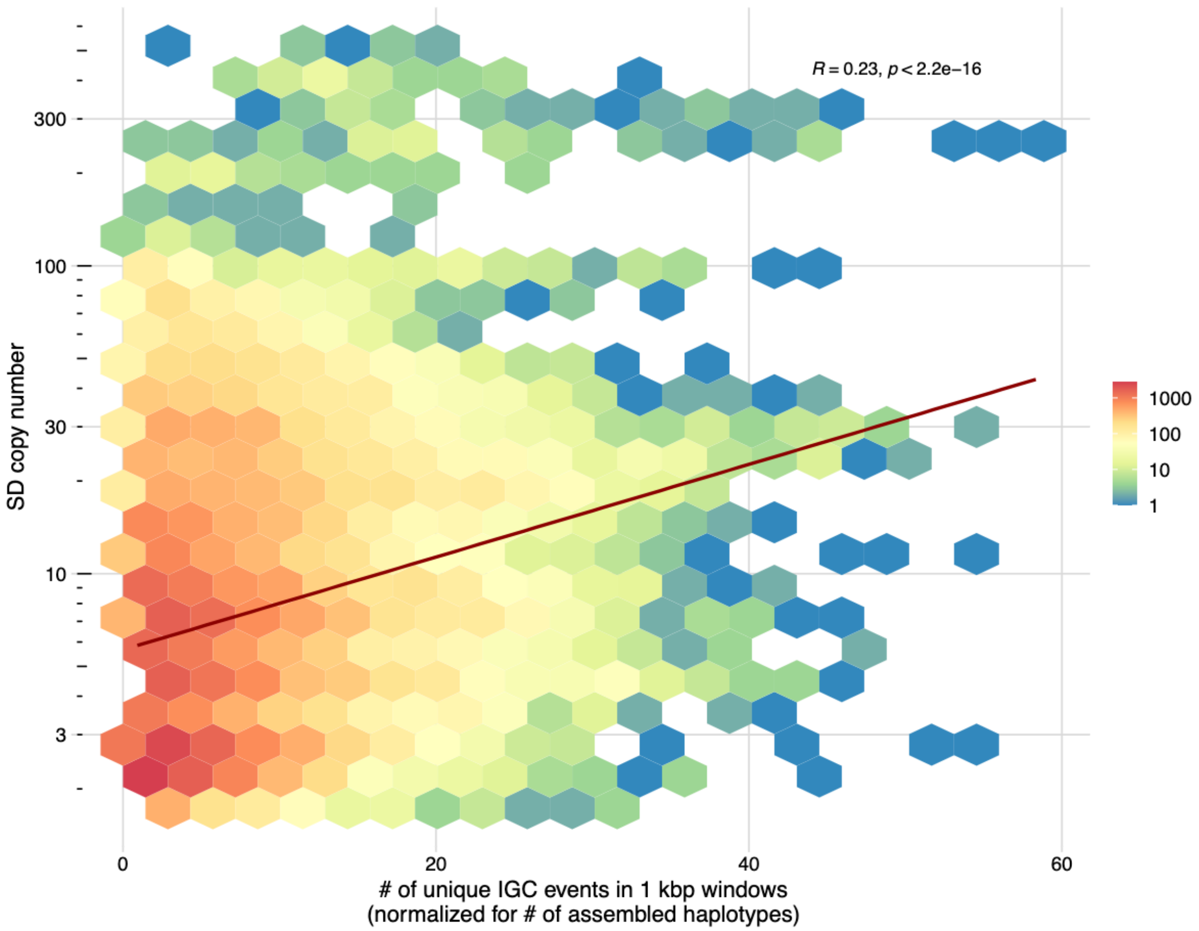

Figure S16. Correlation between the log of the SD copy number and the number of unique IGC events. Shown is the correlation between SD copy number and the number of unique IGC events in a 1 kbp window normalized for the number of assembled haplotypes over that window. The text indicates the value of the Pearson's correlation coefficient and the p-value from a two sided t-test without adjustment for multiple comparisons.

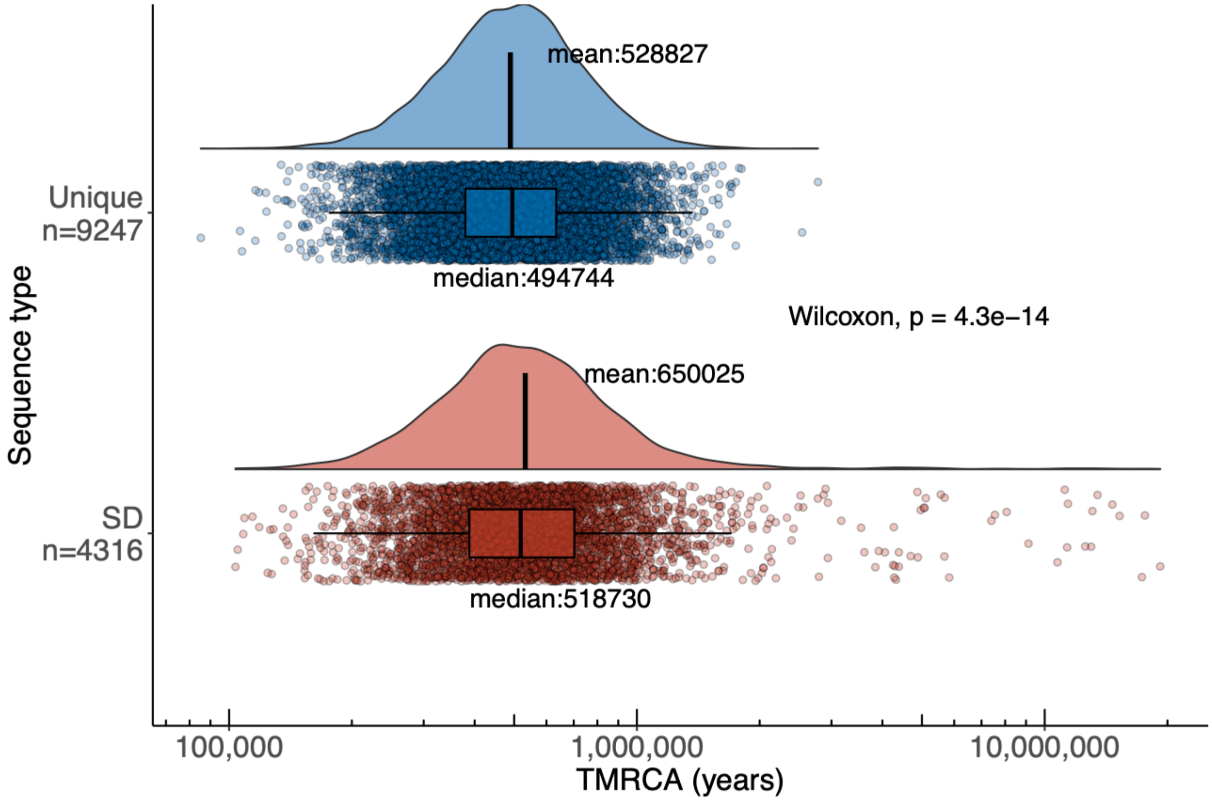

Figure S17. Distributions of time to the most recent common ancestor (TMRCA) for unique (top) and SD (bottom) regions. Measurements are based on nonoverlapping 10 kbp windows from unique (n=9,247 independent windows) and SD (n=4,316 independent windows) sequence. The boxes indicate the range between the first and third quartiles, with the middle line indicating the median. The whiskers show the minimum and maximum value of the data that is within 1.5 times the interquartile range extending from the first and third quartiles, respectively. The p-value is calculated from a one-sided Wilcoxon rank sum test.

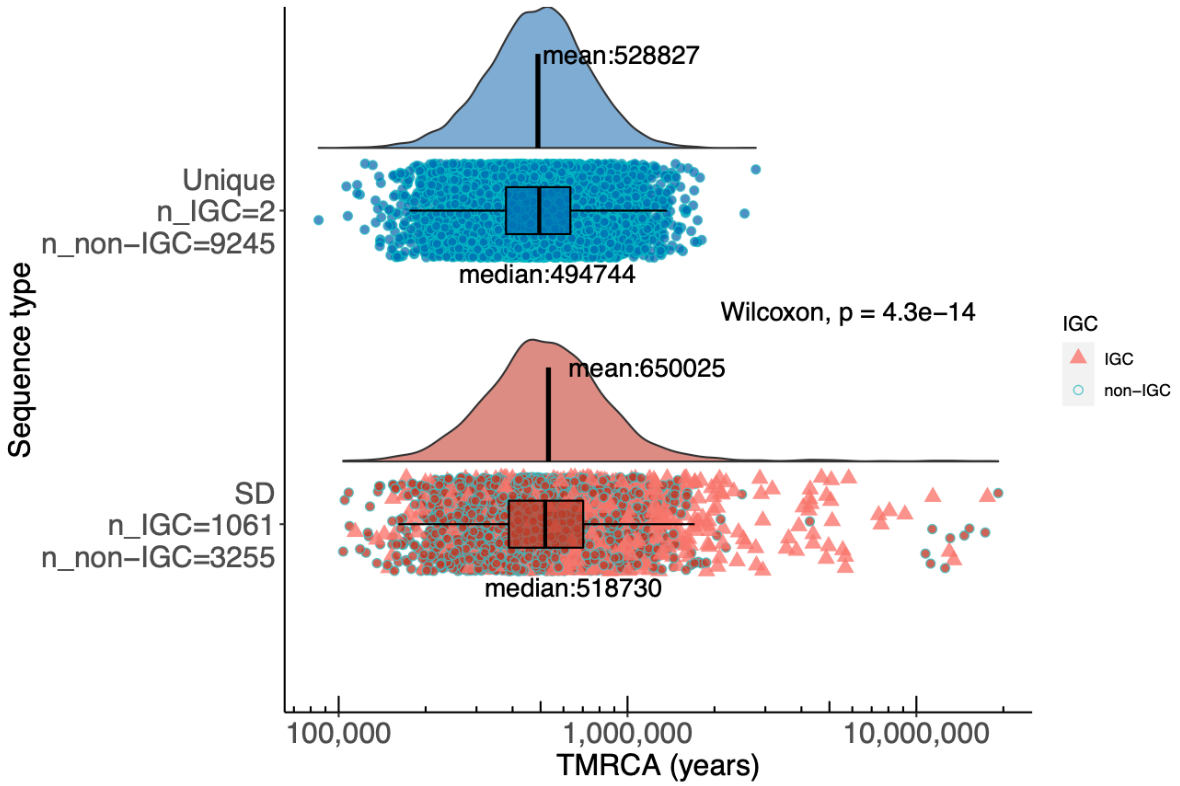

Figure S18. TMRCA distributions for unique (top) and SD (bottom) regions. Measurements are based on nonoverlapping 10 kbp windows from unique (n=9,247 independent windows) and SD (n=4,316 independent windows) sequence. IGC sequences are marked as triangles. The boxes indicate the range between the first and third quartiles, with the middle line indicating the median. The whiskers show the minimum and maximum value of the data that is within 1.5 times the interquartile range extending from the first and third quartiles, respectively. The p-value is calculated from a one-sided Wilcoxon rank sum test.

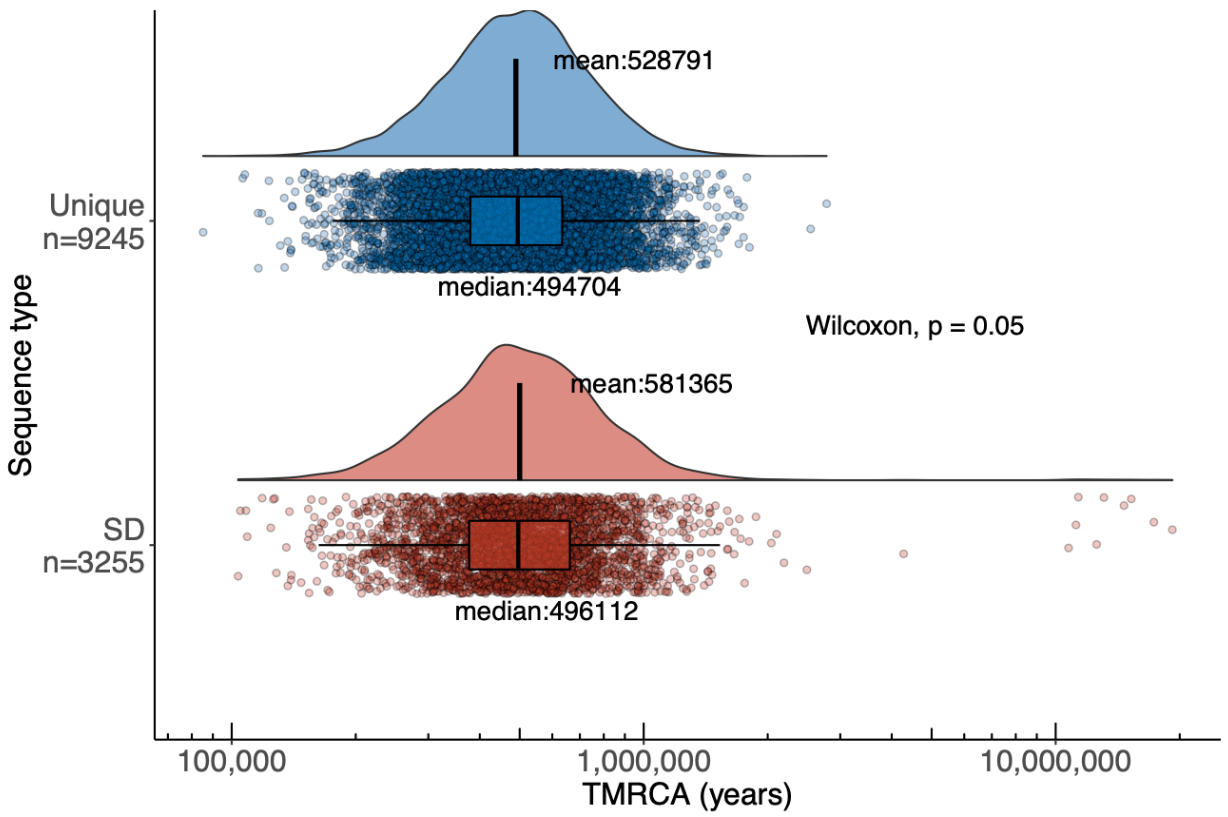

Figure S19. TMRCA distributions for unique (top) and SD (bottom) regions after excluding sequences affected by IGC. Measurements are based on nonoverlapping 10 kbp windows from unique (n=9,245 independent windows) and SD (n=3,255 independent windows) sequence. The boxes indicate the range between the first and third quartiles, with the middle line indicating the median. The whiskers show the minimum and maximum value of the data that is within 1.5 times the interquartile range extending from the first and third quartiles, respectively. The p-value is calculated from a one-sided Wilcoxon rank sum test.

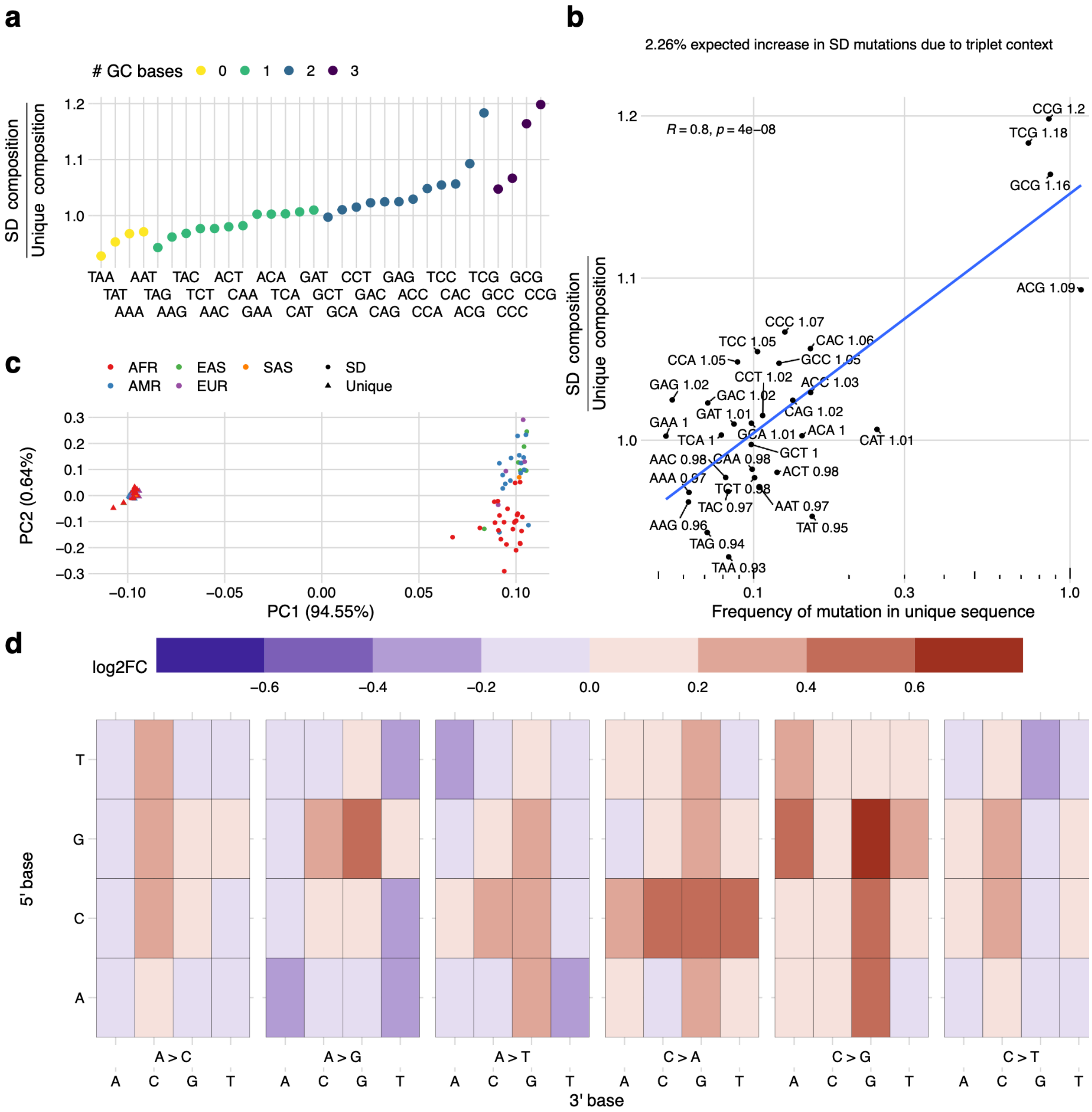

Figure S20. Sequence composition and mutational spectra of SNVs in SDs without IGC.
a) Compositional increase in GC-containing triplets (3-mers) in SDs without IGC versus unique regions of the genome (colored by GC content). **b)** Correlation between the enrichment of certain triplets in SDs compared to the mutability of that triplet in unique regions of the genome. Mutability is defined as the sum of all SNVs that change a triplet divided by the total count of that triplet in the genome. The enrichment ratio of SD over unique is indicated in text next to each triplet sequence. The text (upper left) indicates the value of the Pearson's correlation coefficient and the p-value from a two sided t-test without adjustment for multiple comparisons. **c)** PCA of the mutational spectra of triplets in SD (circles) vs. unique (triangles) regions polarized against a chimpanzee genome assembly and colored by the continental superpopulation of the sample. **d)** Log-fold change between triplet mutation frequency in SD and unique sequences. The y-axis represents the 5' base of the triplet context, the first level of the x-axis shows which central base has changed and how, the second level of the x-axis shows the 3' base, and color represents the log-fold change. For example, the top left corner shows the log-fold change in frequency of TAA > TCA mutations in SD vs. unique sequences.

Supplementary Notes

Quality of HPRC SD content. Compared to previous genome assemblies focused on human diversity [4], Human Pangenome Reference Consortium (HPRC) assemblies of SD regions are significantly more contiguous [5]. However, SDs are frequently a source of misassembly, so we performed a number of validation experiments to further assess the quality of HPRC SDs. Using the T2T reference as a guide of completeness and the HPRC callset of potentially unreliable regions [5], we determined that, on average, only 1.64 Mbp (1.37%) of the analyzed SD sequence was suspect due to abnormal read coverage.

First, we selected 19 copy number variable duplicated loci and compared Illumina WGS read-depth estimates to that predicted by k-merized counts based on summing the two haplotypes from each of the 47 individuals present in the HPRC phase one assemblies (Methods). We observe a striking correlation between the two (r2=0.97) with 756 of 893 tests predicting the exact same copy number (Fig. S1), which suggests that the vast majority of SD gene structures are haplotype resolved with appropriate copy number within HPRC samples. Over half of the failed copy number estimate comparisons occurred within SDs that span greater than 141 kbp and are more than 99.3% identical on average. These constitute some of the largest and most identical SDs in the human genome where extensive structural variation exists.

As a second test for haplotype integrity, we aligned orthogonal ONT sequencing data produced from the same samples. While less accurate than HiFi data, ONT data has the advantage that it is on average three times longer allowing large repetitive regions to be effectively spanned and scaffolded [6]. We devised a strategy to phase [7] and align reads based on matching SUNKs and their distance between the assemblies and the ONT data [8]. We applied this method to assemblies with ultra-long ONT data available (n=35) and, in particular, to IGC acceptor tracts. On average, 94% of acceptor tracts validate by ONT inter-SUNK distances, with an interquartile distance of 94-98%. For 52 large, duplicated loci, we found that 95.9% of the base pairs validate by inter-SUNK distances in ONT reads. While gaps remain and are preferentially enriched in regions of the largest and most identical SDs [9], these analyses confirm haplotype contiguity allowing for patterns of single-nucleotide variation to be assessed for the first time.

As a final control for potential haplotype mixing of long reads from diploid samples confounding SNV diversity analyses, we generated deep ONT and HiFi data from a second hydatidiform mole where a single paternal haplotype was present. Using an alternate assembler (Verkko) that leverages both HiFi and ONT data, we generated a highly contiguous (contig N50 = 110.90 Mbp) and highly accurate (QV = 55) assembly of another haploid human genome [10]. SDs were predictably more contiguous in this haploid sample when compared to the HPRC assemblies though the difference was modest. In the CHM1 assembly we identify an additional 11.9 Mbp of 1:1 sequence alignment (total of 132 Mbp) for SDs when compared to the HPRC hifiasm assemblies (132,082,329-120,190,200 bp, Fig. 1a). We used this second hydatidiform mole as an internal control for all analyses focused on SNV diversity and mutation rate analyses (below). Our analysis showed a more complete assembly of CHM1 did not impact our observations of increased mutational rate across any category. CHM1 has, on average, 12.55 SNVs per 10 kbp, which is in agreement with all the other non-African samples (Fig. 1d). Furthermore, the Mbp of IGC as well as the total number of events in CHM1 (6.69 Mbp, 1,118 events) are very comparable to the other haplotype assemblies [7.47 Mbp, 1,192 events (6.04 Mbp in Europeans)].

Limitations in detecting IGC. There are several assumptions and potential biases in our approach for identifying IGC: 1) the donor sequence must be present in the reference genome for our method to detect IGC, 2) we limit our search to SDs within large (>1 Mbp) 1:1 alignments and therefore may miss IGC events in copy number polymorphic regions, 3) our methods are tuned for finding large (>1 kbp) tracts of IGC within large (>1 kbp) and highly identical (>90%) duplications and are therefore poorly suited to identifying IGC within smaller common repeat elements (e.g., SINE/Alu elements) outside of SDs, and 4) many regions are not assembled well enough to meet our minimum contig length (1 Mbp) resulting in the exclusion of most acrocentric sequence, rDNA clusters, the Y chromosome, centromeres, and human satellite arrays. For these reasons we caution that our subsequent findings on IGC are strictly limited to SDs and are very likely to be an underestimate of the full extent of IGC.

GENECONV validation. To assess the performance of our IGC-calling method, we chose to apply an alternative procedure using a model-based approach implemented in the program GENECONV (v.1.81a) for IGC detection [2,3]. Briefly, GENECONV identifies pairs of uninterrupted sequences with nearly 100% sequence identity that are longer than expected given the overall pattern of variable sites in an alignment. We first identify paralogous sequences and align them using MAFFT (v7.453; mafft --maxiterate 100 sequences.fasta > aln.fasta) and run GENECONV using the following command: geneconv aln.fasta -Annotate -Minnpoly=1 -nolog /lp. To be conservative, we only considered IGC tracts that have both simulation and Karlin-Altschul p-values < 0.05 reported by GENECONV. We applied this approach to re-examine the *TCAF* locus on chromosome 7q35, which has been shown to have wide-spread IGC events in the human genome [11]. In total, GENECONV identifies 87 potential IGC events. Of note, 15 out of 18 highly supported IGC loci (at least 20 supporting SNVs) identified by our alignment-based method are also identified in the 87 GENECONV-based IGC loci.

While the patterns we observe are most consistent with IGC, we recognize that other mutational processes may be at play. To further evaluate our alignment-based IGC calls, we also performed a genome-wide IGC analysis using GENECONV (v1.81) [3]. Briefly, we selected candidate alignment-based IGC loci mapped to reference genome (T2T v1.1) using minimap2 (v2.24-r1122; minimap2 -ax asm20 -t 4 -f 100000000 -N 100 -p 0.3 --eqx -K 100M) and extracted the homologous sequence from each assembly. Multiple sequence alignments were constructed using MAFFT (v7.453) with default settings applied to generate the input for GENECONV. In total, GENECONV identified 22,263 instances of significant IGC calls (nominal p-value <0.05) that overlap with the 25,858 alignment-based IGC loci among the haploid assemblies that we can confidently perform multiple sequence alignment; however, the overlap is redundant and does not represent 1-to-1 mapping. We noticed that GENECONV IGC calls tend to be shorter, and thus, more fragmented over our alignment-based IGC loci (Fig. S13a). In total, there are 8,419 nonredundant GENECONV IGC loci, of which, 7,515 of them have Bonferroni-corrected p-values < 0.05. When compared to our alignment-based IGC loci, there is 29.1% (7,515/25,858) overlap with support from the GENECONV-based analysis (Fig. S13b). It is known that GENECONV has reduced sensitivity for regions with fewer mismatches and, therefore, underestimates the true rate [12]. Consistent with the lack of power of GENECONV on detecting events with fewer mismatches, we found that the number of supporting SNVs from the overlapping calls (mean support SNVs: 56.7) are significantly greater than those from the alignment-based only calls (mean support SNVs: 33.9) (Fig. S13c).

SNV calling with assembly-based methods. SNV calling with hifiasm has been shown to be highly accurate and specific. There are several key papers that have called genetic variants using long-read assemblies although the emphasis initially was on SV calls instead of SNVs [4,13–16]. Later papers, however, have convincingly shown that HiFi accuracy rivals (and in some regions) exceeds Illumina. For example, an analysis using Dipcall from Li et al. [14] estimated the number of false positives per million bases in HiFi assembly-based variant calling to be less than 15 using considerably less accurate long-read data. The number of SNVs called by Illumina and HiFi largely overlap. For example, a comparison of our callset against the NYGC high-coverage Illumina GATK callset for the same sample, HG00733, shows hifiasm calls share over 3.6 million SNVs out of the 3.85 million SNVs seen in the GATK for a recall of 0.933 (Fig. S5). There are an additional 464,506 calls, which are only seen in the hifiasm callset, and overall, the callsets have a Jaccard Similarity Index of 0.833. Out of these 464,506, about half (211,121) occur in tandem repeats where alignments can be ambiguous and many map to GC-rich regions of the genome that are not readily accessible by Illumina sequencing. We also note that we exclude SNVs in tandem repeats from our observations. More recent hifiasm-phased assemblies have been used to extend the gold standard Genome in a Bottle (GIAB) benchmark into more difficult regions while identifying errors in the previous benchmark based on read alignments [17,18]. Finally, the most recently published genome-wide trio-hifiasm shows 99.4% true positive rate for SNVs against the GIAB v4.2.1 benchmark for HG002 [19].

Patterns and QC of single-nucleotide variation in SDs and IGC. Across SD space, the mutational spectrum is the same regardless of position relative to the edge of the SD event. To compute this, we measured the distance between an SNV and the nearest edge of the SD event in which it is contained and divided that by the total length of the SD. Along this scale, 0.5 would represent the exact middle of an SD, and 0.0 would represent an event right on the edge. We observe a uniform distribution across SD length with a small bump between 0.3 and 0.4 observed in all events. This bump, however, is within what we would consider random variability (Fig. S6).

The Ti/Tv ratio is consistent across IGC windows, regardless of the number of supporting SNVs (Fig. S7) with a mean of 1.83 and standard deviation of 0.09 below 100 support SNVs. Above 100 SNVs, the mean is lower (1.66) and standard deviation is higher (0.48) likely due to less genomic space being represented in these bins. The Ti/Tv ratio for SNVs in SDs without IGC for all samples is 1.79, which is generally lower than genome-wide estimates closer to 2. However, we do not include tandem repeats in either SDs or unique space leading to an incomplete sampling of SNV Ti/Tv ratio. We also note that it is not unusual for large stretches of the genome to have Ti/Tv ratios that deviate from 2.0, for example, across all of chromosome 16 (including unique regions) we observe a Ti/Tv of 1.72.

To measure clustering of C>G mutations, we binned the SNV events into six different groups according to their nucleotide change, ignoring direction (e.g., C<->G, A<->T, etc.) and calculated the distance between events of the same type in SD space as a whole, SD space with IGC events, and SD space without IGC events. We observed that median length between events of the same type were consistent across all genomic region categories (Fig. S8).

Finally, we tested the allele frequency distributions of SNVs in SDs and find that it does not vary from the allele frequency of SNVs seen in unique sequence (Fig. S9).

Loss-of-function (LoF) variants in SD genes. We call 23,887 LoF variants across the 50 samples [47 HPRC and 3 Human Genome Structural Variant Consortium (HGSVC) samples], based on Variant Effect Predictor (VEP) predictions of "HIGH" consequence (Online Tables 1-2 [20]). GnomAD v3.1 (hg38 lifted over to CHM13-T2T v2.0) has LoF variant calls for 6,881 (28.8%) of these alleles; 9.9% of HPRC LoF variants in SDs are represented in gnomAD, compared to 39.6% for the remainder of the genome. Among protein-coding genes with an LoF allele count of at least 100 in HPRC, 33/83 (39.8%) are in SDs (Online Table 2 [20]). We observe LoF variants in 83 genes without pLI estimates in gnomAD v2.1, 25 of which are in SDs (30.1%) (Online Table 3 [20]).

To address if there are any paralogous sequence variants in gnomAD that are misclassified, we examined the high-frequency LoF variants in gnomAD and compared to our callset, because there is no known database of paralogous sequence variants. Of the 742 LoF variants in gnomAD with allele frequency of at least 0.3, 292 (39.4%) are never seen in HPRC assemblies (Online Table 4 [20]). The gnomAD inbreeding coefficient/excess heterozygosity filter would remove 106 of these variants from routine analysis, but 167 (57.2%) are unfiltered by gnomAD. Of the 292 high-frequency gnomAD LoF variants never seen in HPRC samples, 101 (34.6%) are in SDs and may correspond to paralogous sequence variants miscalled as LoF variants in a separate paralog in gnomAD, contaminating variant interpretation.

Online Data

Online Tables. Online tables 1-4 are available on Zenodo at https://doi.org/10.5281/zenodo.6792653.

Supplementary References

1. 1000 Genomes Project Consortium *et al.* A map of human genome variation from population-scale sequencing. *Nature* **467**, 1061–1073 (2010).
2. Sawyer. GENECONV: a computer package for the statistical detection of gene conversion. *http://www. math. wustl. edu/~ sawyer*.
3. Sawyer, S. Statistical tests for detecting gene conversion. *Mol. Biol. Evol.* **6**, 526–538 (1989).
4. Ebert, P. *et al.* Haplotype-resolved diverse human genomes and integrated analysis of structural variation. *Science* **372**, (2021).
5. Liao, W.-W. *et al.* A Draft Human Pangenome Reference. *bioRxiv* (2022).
6. Logsdon, G. A. *et al.* The structure, function and evolution of a complete human chromosome 8. *Nature* **593**, 101–107 (2021).
7. Koren, S. *et al.* De novo assembly of haplotype-resolved genomes with trio binning. *Nat. Biotechnol.* **36**, 1174–1182 (2018).
8. Dishuck, P. C., Rozanski, A. N., Logsdon, G. A., Porubsky, D. & Eichler, E. E. GAVISUNK: genome assembly validation via inter-SUNK distances in Oxford Nanopore reads. *Bioinformatics* **39**, btac714 (2022).
9. Porubsky, D. *et al.* Gaps and complex structurally variant loci in phased genome assemblies. *bioRxiv* 2022.07.06.498874 (2022) doi:10.1101/2022.07.06.498874.
10. Rautiainen, M. *et al.* Telomere-to-telomere assembly of diploid chromosomes with Verkko. *Nat. Biotechnol.* (2023) doi:10.1038/s41587-023-01662-6.
11. Hsieh, P. *et al.* Evidence for opposing selective forces operating on human-specific duplicated TCAF genes in Neanderthals and humans. *Nat. Commun.* **12**, 5118 (2021).
12. Mansai, S. P. & Innan, H. The power of the methods for detecting interlocus gene conversion. *Genetics* **184**, 517–527 (2010).
13. Huddleston, J. *et al.* Discovery and genotyping of structural variation from long-read haploid genome sequence data. *Genome Res.* **27**, 677–685 (2017).
14. Li, H. *et al.* A synthetic-diploid benchmark for accurate variant-calling evaluation. *Nat. Methods* **15**, 595–597 (2018).
15. Vollger, M. R. *et al.* Improved assembly and variant detection of a haploid human genome using single-molecule, high-fidelity long reads. *Ann. Hum. Genet.* **84**, 125–140 (2020).
16. Heller, D. & Vingron, M. SVIM-asm: Structural variant detection from haploid and diploid genome assemblies. *Bioinformatics* (2020) doi:10.1093/bioinformatics/btaa1034.
17. Chin, C.-S. *et al.* A diploid assembly-based benchmark for variants in the major histocompatibility complex. *Nat. Commun.* **11**, 4794 (2020).
18. Wagner, J. *et al.* Curated variation benchmarks for challenging medically relevant

autosomal genes. *Nat. Biotechnol.* **40**, 672–680 (2022).

19. Jarvis, E. D. *et al.* Semi-automated assembly of high-quality diploid human reference genomes. *Nature* **611**, 519–531 (2022).
20. Vollger, M. Supplemental data for: Increased mutation and gene conversion within human segmental duplications. Preprint at https://doi.org/10.5281/ZENODO.6792653 (2023).
