# Supplementary material for: Increased mutation and gene conversion within human segmental duplications
Source: Nature. 2023 May 10;617(7960):325–34. doi: 10.1038/s41586-023-05895-y (PMC10172114; doi:10.1038/s41586-023-05895-y)
Supplement: Supplementary file 2 — Reporting Summary [file 41586_2023_5895_MOESM2_ESM.pdf]

nature portfolio

Corresponding author(s): Evan E Eichler

Last updated by author(s): Feb 19, 2023

Reporting Summary

Nature Portfolio wishes to improve the reproducibility of the work that we publish. This form provides structure for consistency and transparency in reporting. For further information on Nature Portfolio policies, see our Editorial Policies and the Editorial Policy Checklist.

Statistics

For all statistical analyses, confirm that the following items are present in the figure legend, table legend, main text, or Methods section.

n/a	Confirmed	
☐	☒	The exact sample size (*n*) for each experimental group/condition, given as a discrete number and unit of measurement
☒	☐	A statement on whether measurements were taken from distinct samples or whether the same sample was measured repeatedly
☐	☒	The statistical test(s) used AND whether they are one- or two-sided<br>*Only common tests should be described solely by name; describe more complex techniques in the Methods section.*
☐	☒	A description of all covariates tested
☐	☒	A description of any assumptions or corrections, such as tests of normality and adjustment for multiple comparisons
☐	☒	A full description of the statistical parameters including central tendency (e.g. means) or other basic estimates (e.g. regression coefficient) AND variation (e.g. standard deviation) or associated estimates of uncertainty (e.g. confidence intervals)
☐	☒	For null hypothesis testing, the test statistic (e.g. *F*, *t*, *r*) with confidence intervals, effect sizes, degrees of freedom and *P* value noted<br>*Give P values as exact values whenever suitable.*
☒	☐	For Bayesian analysis, information on the choice of priors and Markov chain Monte Carlo settings
☒	☐	For hierarchical and complex designs, identification of the appropriate level for tests and full reporting of outcomes
☐	☒	Estimates of effect sizes (e.g. Cohen's *d*, Pearson's *r*), indicating how they were calculated

Our web collection on statistics for biologists contains articles on many of the points above.

Software and code

Policy information about availability of computer code

Data collection	No software was used.

Data analysis

The software pipeline for aligning assemblies and calling IGC is available on GitHub (https://github.com/mrvollger/asm-to-reference-alignment v0.1) and Zenodo (https://zenodo.org/record/7653446). Code for analyzing variants called against T2T-CHM13 v1.1 is available on GitHub (https://github.com/mrvollger/sd-divergence v0.1) and Zenodo (https://zenodo.org/record/7653464). The software pipeline for analyzing the triple context of SNVs is available on GitHub (https://github.com/mrvollger/mutyper_workflow v0.1) and Zenodo (https://zenodo.org/record/7653472). Scripts for figure and table generation are available on GitHub (https://github.com/mrvollger/sd-divergence-and-igc-figures v0.1) and Zenodo (https://zenodo.org/record/7653486). GAVISUNK is available on GitHub (https://github.com/pdishuck/GAVISUNK) and Zenodo (https://zenodo.org/record/7655335).

The custom pipelines listed above take advantage of additional tools which include:
- mutyper=0.6.1
- bcftools=1.13
- bedtools=2.30
- dipcall=0.3
- minimap2=2.24
- pysam=0.19.1
- rustybam=0.1.29
- samtools=1.13
- flagger=0.1
- gavisunk=1.0

For manuscripts utilizing custom algorithms or software that are central to the research but not yet described in published literature, software must be made available to editors and reviewers. We strongly encourage code deposition in a community repository (e.g. GitHub). See the Nature Portfolio guidelines for submitting code & software for further information.

Data

Policy information about availability of data

All manuscripts must include a data availability statement. This statement should provide the following information, where applicable:

- Accession codes, unique identifiers, or web links for publicly available datasets
- A description of any restrictions on data availability
- For clinical datasets or third party data, please ensure that the statement adheres to our policy

PacBio HiFi and ONT data have been deposited into NCBI Sequence Read Archive (SRA) under the following BioProject IDs: PRJNA850430, PRJNA731524, PRJNA551670, PRJNA540705, and PRJEB36100. PacBio HiFi data for CHM1 are under the following SRA accessions: SRX10759865 and SRX10759866. Sequencing data for Clint PTR is available on NCBI SRA under the BioProject PRJNA659034. The T2T-CHM13 v1.1 assembly can be found on NCBI (GCA_009914755.3). Cell lines obtained from the NIGMS Human Genetic Cell Repository at the Coriell Institute for Medical Research are listed in Table S1. Assemblies of HPRC samples are available on NCBI under the BioProject PRJNA730822. All additional assemblies used in this work (Clint PTR, CHM1, HG00514, NA12878, HG03125), variants calls, assembly alignments, and other annotation data used in analysis are available on Zenodo (https://doi.org/10.5281/zenodo.6792653).

Human research participants

Policy information about studies involving human research participants and Sex and Gender in Research.

Reporting on sex and gender

The biological sex (male and female) of the samples used has been included; however, variation on the Y chromosome was not accessed due to an incomplete reference assembly.

Population characteristics

The superpopulation of individuals has been included.

Recruitment

Participants were recruited in separate studies from this study.

Ethics oversight

Sample were collected by other studies as part of the 1000 Genomes Project with the following consent form: https://www.internationalgenome.org/sites/1000genomes.org/files/docs/Informed%20Consent%20Form%20Template.pdf

Note that full information on the approval of the study protocol must also be provided in the manuscript.

Field-specific reporting

Please select the one below that is the best fit for your research. If you are not sure, read the appropriate sections before making your selection.

☒ Life sciences ☐ Behavioural & social sciences ☐ Ecological, evolutionary & environmental sciences

For a reference copy of the document with all sections, see nature.com/documents/nr-reporting-summary-flat.pdf

Life sciences study design

All studies must disclose on these points even when the disclosure is negative.

Sample size	In this study we used all available samples with comparable assembly quality, including 47 samples from the HPRC, 3 samples from HGSVC, and the haploid assemblies of CHM1, CHM13, and GRCh38.
Data exclusions	No data excluded.
Replication	All analysis can be replicated using the software pipelines posted on GitHub and Zenodo.
Randomization	The allocation of samples was not random as we used all available samples. Furthermore, it was not necessary as we did not perform analysis comparing cases versus controls.
Blinding	Blinding is not applicable to this study because we did not perform any experiments where there was treatment and control groups that would necessitate blinding.

Reporting for specific materials, systems and methods

We require information from authors about some types of materials, experimental systems and methods used in many studies. Here, indicate whether each material, system or method listed is relevant to your study. If you are not sure if a list item applies to your research, read the appropriate section before selecting a response.

Materials & experimental systems

n/a	Involved in the study	
☒	☐	Antibodies
☐	☒	Eukaryotic cell lines
☒	☐	Palaeontology and archaeology
☒	☐	Animals and other organisms
☒	☐	Clinical data
☒	☐	Dual use research of concern

Methods

n/a	Involved in the study	
☒	☐	ChIP-seq
☒	☐	Flow cytometry
☒	☐	MRI-based neuroimaging

Eukaryotic cell lines

Policy information about cell lines and Sex and Gender in Research

Cell line source(s)	CHM13hTERT (abbr. CHM13) cells were originally isolated from a hydatidiform mole at Magee-Womens Hospital (Pittsburgh, PA) as part of a research study (IRB MWH-20-054). All other transformed lymphoblast cell lines belonging to the 1000 Genomes Project were obtained from the Coriell Cell Repository as part of the NHGRI catalog. Cell lines obtained from the NIGMS Human Genetic Cell Repository at the Coriell Institute for Medical Research are listed in Table S1.
Authentication	The CHM13hTERT cell line was authenticated via STR analysis and karyotyped to show a 46,XX karyotype (Miga et al., Nature, 2020). The other cell lines used in this study have not been authenticated to our knowledge.
Mycoplasma contamination	The CHM13hTERT cell line is negative for mycoplasma contamination (Miga et al., Nature, 2020). The other cell lines used in this study have not been assessed for mycoplasma contamination to our knowledge.
Commonly misidentified lines (See ICLAC register)	No commonly misidentified cell lines were used in this study.
